# Titin activates myosin filaments in skeletal muscle by switching from an extensible spring to a mechanical rectifier

**DOI:** 10.1073/pnas.2219346120

**Published:** 2023-02-22

**Authors:** Caterina Squarci, Pasquale Bianco, Massimo Reconditi, Irene Pertici, Marco Caremani, Theyencheri Narayanan, Ádám I. Horváth, András Málnási-Csizmadia, Marco Linari, Vincenzo Lombardi, Gabriella Piazzesi

**Affiliations:** ^a^PhysioLab, University of Florence, 50019 Firenze, Italy; ^b^European Synchrotron Radiation Facility – The European Synchrotron, Grenoble 38043, France; ^c^Magyar Tudományos Akadémia - Eötvös Loránd University Motor Pharmacology Research Group 1117, Budapest, Hungary; ^d^Motorpharma, Ltd. 1026, Budapest, Hungary

**Keywords:** titin, myosin filament activation, skeletal muscle, striated muscle, muscle regulation

## Abstract

Titin is a molecular spring in parallel with myosin motors in each muscle half-sarcomere, responsible for passive force development at sarcomere length (SL) above the physiological range (>2.7 μm). The role of titin at physiological SL is unclear and is investigated here in single intact muscle cells of the frog (*Rana esculenta*), by combining half-sarcomere mechanics and synchrotron X-ray diffraction in the presence of 20 μM para-nitro-blebbistatin, which abolishes the activity of myosin motors and maintains them in the resting state even during activation of the cell by electrical stimulation. We show that, during cell activation at physiological SL, titin in the I-band switches from an SL-dependent extensible spring (OFF-state) to an SL-independent rectifier (ON-state) that allows free shortening while resisting stretch with an effective stiffness of ~3 pN nm^−1^ per half-thick filament. In this way, I-band titin efficiently transmits any load increase to the myosin filament in the A-band. Small-angle X-ray diffraction signals reveal that, with I-band titin ON, the periodic interactions of A-band titin with myosin motors alter their resting disposition in a load-dependent manner, biasing the azimuthal orientation of the motors toward actin. This work sets the stage for future investigations on scaffold and mechanosensing-based signaling functions of titin in health and disease.

## Significance

Skeletal and cardiac myopathies are often associated with mutations in the giant, cytoskeleton protein titin, but the role of titin during contraction at physiological sarcomere length is not known. Here, we demonstrate a new mechanism by which titin stiffness increases by orders of magnitude upon cell activation. This mechanism enables titin to trigger the mechanosensing-based activation of the myosin motors that drives them to interact with the overlapping actin filaments. This paper sets the stage for future studies in demembranated cells from both mammalian models and human biopsies, aimed at defining the genotype-phenotype relation of titin variants in health and disease and developing specific therapeutical strategies.

**Fig. 1.**
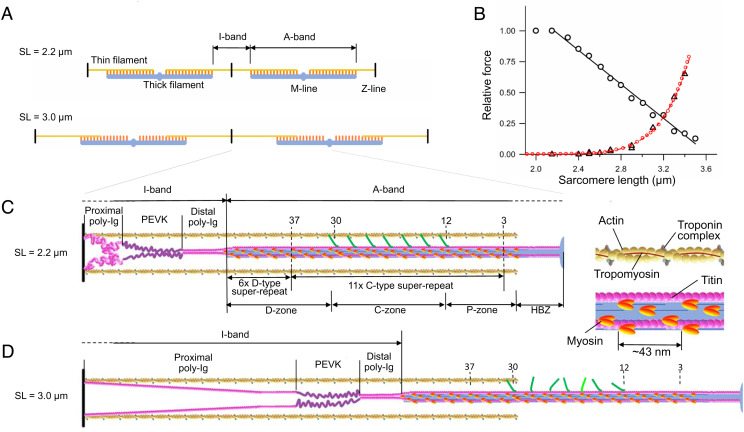
Structure–function of myofilaments and titin in relation to the length of the sarcomere. (*A*) Overview of the thick filament (blue), myosin motors (orange), and thin filament (yellow) at SL 2.2 μm (full overlap) and 3.0 μm (partial overlap). (*B*) Relation between SL and either active force at the plateau of the isometric tetanic contraction (black circles, linear fit to points at SL >2.2 μm, continuous line) or passive force (triangles, fitted with an exponential equation (red dashed line) and with the model (red circles) described in *SI Appendix*, *Supporting Note* 1 and Fig. S1). Data from ref. 2. (*C*) Protein disposition on the thin (yellow) and thick (light blue) filaments in the half-sarcomere at rest at 2.2 μm SL. M-line on the right and Z-line on the left. *Inset*: Overlap region on an enlarged scale to show with better resolution the ~38 nm axial periodicity of the troponin complex (gray) along the thin filament and the two motor domains (orange) of each myosin molecule tilted back on their tail (blue) in the OFF state (3, 4). The 49 crowns of motors are numbered starting from the M-line; thin filament with tropomyosin (brown) and troponin complex (gray); MyBP-C (green) aligned with myosin triplets from crowns 12 to 30. Titin (magenta) with PEVK segment identified by dark magenta. HBZ, half-bare zone. P-, C-, and D-zones, proximal, MyBP-C containing and distal zones. Only two of the six titin molecules and only one of the three series of MyBP-C molecules per htf are represented for clarity. (*D*) Straightening of the proximal tandem Ig segment by passive stretch to 3.0 μm SL.

Contraction of the striated muscle is powered by the cyclical adenosine triphosphate (ATP)-fueled interactions of the motor protein myosin II, arranged in two bipolar arrays on thick filaments originating at the midpoint of each sarcomere (M-line), with the nearby thin, actin-containing filaments originating at the sarcomere extremities (Z-line, Fig. 1*A*). In the half-sarcomere, myosin motors are mechanically coupled as parallel force generators and the collective force depends on the number of motors available for actin attachment and thus on the degree of overlap between thick and thin filaments (Fig. 1*B*, black circles; ref. 1). The half-sarcomere is the basic functional unit in which the emergent properties from the arrays of myosin motors, the interdigitating thin filaments, and a “third” filament made by the cytoskeleton protein titin (Fig. 1*C*) account for the mechanical performance of muscle and its regulation.

Titin is a giant protein (up to 4 MDa) that spans the half-sarcomere (Fig. 1*C*, magenta), first through the I-band, connecting the Z-line with the tip of the thick filament, and then through the A-band, associated with the thick filament (six molecules per thick filament; refs. 5, 6) up to the M-line at the center of the sarcomere (7–9). The titin I-band region acts as a spring able to transmit the stress also when no myosin motors are attached to actin. In the muscle fiber of the frog, in which there is no contribution from extracellular matrix components (10, 11), titin is responsible for the passive force when the muscle cell is stretched at rest (Fig. 1*B*, triangles; refs. 12–16). Within the I-band titin, the distal tandem immunoglobulin-like segment forms a stiff end-filament composed of the six titin molecules attaching to the tip of the thick filament (17, 18), while the other two segments account for titin extensibility: the proximal tandem Ig segment (hereinafter called tandem Ig segment) and the unique sequence rich in proline (P), glutamate (E), valine (V), and lysine (K) residues (PEVK segment). Both spring-like segments exhibit variable muscle-type specific lengths (7, 19), which account for the differences in passive force–sarcomere length (SL) relations (20). In situ studies using immunofluorescence and immunoelectron microscopy on skinned fibers and myofibrils from mammalian skeletal muscle demonstrated that the large extensibility of the muscle sarcomere at SL < 2.7 μm is enabled by straightening out of randomly bent elements of the tandem Ig segment. At longer SL, at which the tandem Ig segment approaches its contour length, the passive force increases more steeply, reflecting the PEVK segment stiffness (*SI Appendix*, *Supporting Note* 1 and Fig. S1; see refs. 21–25).

Titin in the A-band is composed of Ig and fibronectin (Fn) domains each ~4 nm long, with two distinct domain superrepeats: 11 “C-type” superrepeats, each composed of 11 Ig-Fn domains, extending from about layer 3 to 37 of the myosin crowns, and 6 “D-type” superrepeats, each composed of seven Ig-Fn domains, extending from about layer 38 to the filament tip (Fig. 1*C*; refs. 7, 26–28). The A-band region of titin is made inextensible by its association to the other proteins in the thick filament, myosin, and the Myosin Binding Protein C (MyBP-C), an accessory protein that is bound with its C terminus to the central one-third of the half-thick filament (htf) (C-zone, from layer 12 to 30, Fig. 1*C*) and extends from the thick filament backbone to establish dynamic interactions with the thin filament (2, 29–31) with its N terminus.

I-band titin transmits any pulling force exerted on the extremity of the half-sarcomere to the tip of the thick filament and in this way could play a role in thick filament mechanosensing that switches myosin motors ON (3, 32). Moreover, as an elastic element in parallel with motors, I-band titin could preserve the homogeneity of sarcomeres during contraction, by preventing the lengthening of weak half-sarcomeres. However, titin-dependent passive force typically rises steeply only at SL > 2.6 µm (Fig. 1*B*; refs. 2, 10, 11, 22, 24, 33), and thus I-band titin stiffness is too low for the above functions at physiological SL, unless it gets much larger during contraction.

The mechanical definition of the I-band titin in situ in the active half-sarcomere is hampered by the presence of the in-parallel array of myosin motors with a stiffness that is more than one order of magnitude larger than titin stiffness (34). Here, we use para-nitro-blebbistatin (PNB; ref. 35) to inhibit actin–myosin interaction during tetanic stimulation of a frog muscle cell. In addition, 20 μM PNB suppresses in vitro actin-triggered ATPase activity of frog muscle myosin S1 and heavy meromyosin (HMM) (*SI Appendix*, *Materials and Methods* and Fig. S2 *A* and *B*) and the mechanical response of the muscle cell to tetanic stimulation (*SI Appendix*, Fig. S2 *C*–*E*), maintaining the motors in the OFF-state conformation, in which they lie tilted back on the surface of the thick filament (Fig. 1*C* and *SI Appendix*, Fig. S3) (4, 36). Previous studies noted that blebbistatin does not fully suppress the mechanical response and maintain the motor OFF-state upon Ca^2+^ activation in skinned rabbit psoas fibers (32, 37). This is likely a consequence of either the lower inhibitory power of blebbistatin as compared to PNB (*SI Appendix*, Fig. S2 *A* and *B*) or intrinsic limits of skinned preparations to fully preserve the motor OFF-structure (27, 38).

Here, we used sarcomere-level mechanics and small-angle X-ray fiber diffraction to determine the titin-dependent mechanical and structural responses to a stepwise increase in load imposed on the muscle cell under PNB-inhibitory conditions. Length changes in units of nanometer per half-sarcomere (hereinafter referred to as nm) were measured with a striation follower in a population of ~500 sarcomeres. We discovered that upon stimulation at physiological SL, titin in the I-band switches from the OFF-state characterized by large extensibility to the ON-state in which it exhibits rectifying properties, allowing free shortening, while opposing stretching with a viscosity coefficient three orders of magnitude larger that underpins an effective stiffness of 3 pN nm^−1^. With the I-band titin in the ON-state, the periodic interactions between A-band titin and myosin motors are able to activate the thick filament by perturbing the resting disposition of motors on the surface of the thick filament in a load-dependent manner and biasing them toward the sixfold rotational symmetry of the thin filaments in the myofilament lattice.

## Results

### In the Muscle Cell at Rest, the I-Band Titin Behaves as a Viscoelastic Spring with Large SL-Dependent Extensibility.

**Fig. 2.**
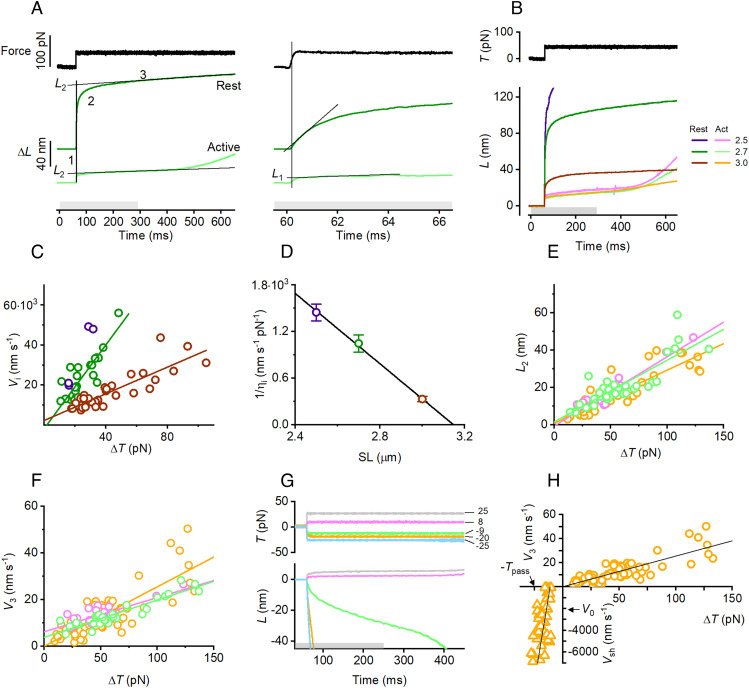
During muscle cell activation titin changes from an extensible spring to a mechanical rectifier resisting to pulling forces and shortening freely under restoring forces. (*A*) Lengthening responses to a positive force step of 50 pN (upper trace, black) imposed either at rest (dark green) or during stimulation (light green) at 2.7 μm SL. The gray horizontal bar indicates the stimulation duration. In the *Right* panel, records are 100 times faster than in the *Left* panel. In both panels, the vertical line marks the half-time of the step. (*B*) Superimposed responses to the same step as in *A* imposed either at rest (dark colors) or during stimulation (light colors) at different SLs indicated by the color code in the *Inset*, where figures indicate the SL (μm): violet and magenta, 2.5 μm; dark and light green, 2.7 μm; brown and orange, 3.0 μm. The same color code is used throughout panels *C*–*F*, and *H*, in which the lines are the linear fit to pooled data unless differently specified. (*C*) *V*_i_*–*Δ*T* relation at rest. (*D*) 1/η_i_–SL relation at rest. (*E*) *L*_2_–Δ*T* relations during stimulation. (*F*) *V*_3_–Δ*T* relation during stimulation. (*G*) Superimposed length changes (lower traces) in response to force steps (upper traces) of different sizes and directions, as indicated by figures close to the force traces (pN), imposed during stimulation (gray bar) at 3 μm SL. (*H*) *V*–Δ*T* relations during stimulation at 3 μm SL. The ordinate is *V*_3_ for the right upper quadrant (same data as orange points in panel *F*) and *V*_sh_ for the left lower quadrant (data for negative force steps). The ordinate scale is 10^2^ larger for negative values. The line in the left lower quadrant is obtained as the average of the linear fits to data from individual fibers (*SI Appendix*, *Materials and Methods*). The arrows indicate the passive force (*T*_pass_) at this SL and the unloaded shortening velocity in control contractions (*V*_0_). Circles, data from 22 fibers; triangles, data from eight fibers.

A force step Δ*T* of 0.2 *T*_0,c_ (the isometric tetanic force developed in the control solution at 4 °C and 2.15 μm SL) imposed on the cell elicits a lengthening response that is much larger at rest (Fig. 2*A*, dark green) than during tetanic stimulation (light green). The response at rest has a fast component (phase 2), the velocity of which decreases exponentially to a steady slow value attained within 200 ms (phase 3), while in the response during tetanic stimulation, phase 2 is smaller and briefer (complete within ~2 ms) and preceded by an elastic component simultaneous with the force step (phase 1, Fig. 2 *A*, *Right* panel, light green).

In the cell at rest, the amplitude of the fast component (*L*_2_, estimated by back-extrapolating to the half-time of the step the tangent to phase 3 lengthening) decreases with the increase of the starting SL (Fig. 2*B* and *SI Appendix*, Fig. S4*A*, dark color traces) and, at a given SL, increases with the step size (*SI Appendix*, Fig. S4 *A*–*C*, dark color traces and symbols): *L*_2_ becomes >120 nm and exceeds the range of movement of the motor (see *SI Appendix*, Supporting Materials and Methods) for a step size that depends on the starting SL: 0.13 *T*_0,c_ at 2.3 μm (*SI Appendix*, Fig. S4 *A*, *Left* panel, dark gray), 0.2 *T*_0,c_ at 2.5 μm (*Middle* panel, violet), and 0.4 *T*_0,c_ at 2.7 μm (*Right* panel, dark green).

The rapid lengthening at rest is characterized by an initial velocity [*V*_i_, estimated by the slope of the tangent to the initial 0.5 ms of the length response following the step end (Fig. 2 *A*, *Right* panel, black line on the dark green trace)], which at any given SL increases with the size of the force step (*SI Appendix*, Fig. S4*B*) and, for a given step size, decreases with the increase of SL [Fig. 2*C*, compare violet (2.5 μm), dark green (2.7 μm), and brown (3.0 μm)]. *T*_0,c_ is 144 ± 18 kPa (mean ± SD, 22 fibers), which, from the lattice geometry of the frog muscle cell (5.87 * 10^14^ thick filaments m^−2^; ref. 39), corresponds to 245 ± 32 pN per htf. Consequently, a force step of ~0.2 *T*_0,c_ corresponds to ~50 pN per htf (hereinafter referred to as pN). The slope of the first-order regression line fitted to the *V*_i_–Δ*T* relations (Fig. 2*C*) represents an estimate of the reciprocal of a viscous coefficient (or fluidity coefficient, 1/η_i_), which is ~10^3^ nm s^−1^ pN^−1^ at 2.7 μm SL (green line in Fig. 2*C*) and decreases to 1/3 this value at 3 μm SL (brown line). At 2.5 μm SL, only small Δ*T* can be used (*SI Appendix*, Fig. S4*A* and Fig. 2*C*, violet), but assuming a direct proportionality between *V*_i_ and Δ*T*, 1/η_i_ can be estimated also at this SL (blue circle in Fig. 2*D*). In the range of the explored SL, 1/η_i_ decreases linearly with increase in SL (Fig. 2*D*). The abscissa intercept of the first-order regression line fitted to data, 3.15 ± 0.02 μm, estimates the SL at which the fluidity coefficient reduces to zero, which likely corresponds to the SL at which the tandem Ig segment stops behaving as an entropic spring attaining its contour length (*SI Appendix*, Fig. S1). Accordingly, at 3 μm SL, *L*_2_ shows a direct proportionality to Δ*T* (*SI Appendix*, Fig. S4*C*, brown circles and line), underpinning an effective compliance of ~0.65 nm pN^−1^ (Table on the right of *SI Appendix*, Fig. S4*C*), likely attributable to the PEVK segment. At 2.7 μm SL, instead, the large viscoelastic extensibility of the tandem Ig segment still adds to that of the PEVK, shifting the relation upward (*SI Appendix*, Fig. S4*C*, green symbols and line).

### Upon Electrical Stimulation of the Muscle Cell the I-Band Titin Becomes a Rigid Spring with an SL-Independent Stiffness.

The amplitude of phase 2 response to a force step imposed during tetanic stimulation (light colors in Fig. 2 *A* and *B* and *SI Appendix*, Fig. S4*A*) is much smaller than that at rest (dark colors). In contrast to the response at rest, *L*_2_ during stimulation is almost independent of SL (Fig. 2*B*, light colors traces). *L*_2_ increases with the step size (*SI Appendix*, Fig. S4 *A* and *D* and Fig. 2*E*) and the slope of the linear fit to the *L*_2_–Δ*T* relation (lines in Fig. 2*E*) does not vary with SL, underpinning a similar effective compliance, the reciprocal of which, the stiffness *e*_2_, is ~3 pN nm^−1^ (Table 1). The phase 1 component of the response simultaneous with the force step (Fig. 2 *A*, *Right*) has an amplitude (*L*_1_, estimated by back-extrapolating to the half-time of the step the tangent to the initial part of phase 2, black line on the light green trace) that is independent of SL and increases in proportion to the step size (*SI Appendix*, Fig. S4 *D* and *E*) supporting an instantaneous half-sarcomere compliance (the slope of the *L*_1_/Δ*T* relation, Table on the right), the reciprocal of which, the stiffness *e*_1_, is ~7 pN nm^−1^ (Table 1). The later slow phase 3 occurs at a speed *V*_3_, which increases with the size of the force step (Fig. 2*F*) suggesting a viscous-like nature of the response. The fluidity coefficient 1/η_3_, estimated by the slope of the linear fit to the *V*_3_–Δ*T* data in Fig. 2*F*, is 0.15 to 0.25 nm s^−1^ pN^−1^, three orders of magnitude smaller than 1/η_i_ in the resting fiber (Fig. 2*D*) and roughly independent of SL. Its reciprocal, the viscosity coefficient η_3_, ranges from 4 to 6 pN s nm^−1^ (Table 1). Following the end of stimulation (290 ms, indicated by the end of the gray bar in Fig. 2 *A* and *B*), there is a sharp transition to a faster lengthening marking the time at which the half-sarcomere extensibility at rest is resumed. The increase in lengthening velocity decreases with SL (*SI Appendix*, Fig. S4*A*), as the tandem Ig segment approaches its contour length.

**Table 1.** Relevant mechanical parameters of the response to force steps imposed on the active fiber at SL 2.5, 2.7, and 3.0 μm

SL (µm)	*e*_1_ (pN nm^−1^)	*e*_2_ (pN nm^−1^)	η_3_ (pN s nm^−1^)	η_sh_ (pN s nm^−1^)
2.5	6.90 ± 0.52	2.69 ± 0.23	6.85 ± 1.13	
2.7	7.30 ± 0.96	3.02 ± 0.33	6.25 ± 0.51	
3.0	7.35 ± 0.65	3.51 ± 0.29	3.94 ± 0.32	(1.60 ± 0.35) × 10^−3^

*e*_1_, *e*_2_ and h_3_ parameters of the lengthening response to a positive force step: *e*_1_ instantaneous stiffness; *e*_2_, stiffness calculated from the relation between *L*_2_ (the amplitude of the fast component of the lengthening transient) and the step size Δ*T* (Fig. 2*E*); η_3_, viscosity coefficient from the *V*_3_-Δ*T* relation (Fig. 2*F*). η_sh_, viscosity coefficient from the *V*_sh_-Δ*T* relation at 3.0 mm SL (triangles in Fig. 2*H*). Data from 22 fibers, *n* = 17 to 69. Errors are SEM. The differences among *e*_1_ and *e*_2_ values at different SL are not statistically significant [*P* > 0.59 (*e*_1_)and > 0.03 (*e*_2_)]. η_3_ at 3 μm SL is significantly different from values at 2.5 and 2.7 μm (*P* < 0.01) and all three values are three orders of magnitude larger than η_sh_. pN refer to force per half thick filament and nm to length changes per half-sarcomere

In conclusion, the large half-sarcomere extensibility exhibited by the resting fiber in the few hundred milliseconds following a sudden increase in load is explained by the entropic contribution of the straightening of the randomly bent elements of the tandem Ig segment that progressively reduces with the increase of SL and becomes zero at ~3.1 μm SL (Fig. 2*D*), at which the tandem Ig segment approaches its contour length. In contrast, in the same SL range, the extensibility of the active fiber is constant and quite small, underpinning an effective stiffness *e*_2_ of ~3 pN nm^−1^ (Table 1). This is explained by the presence of a viscous element with a drag coefficient as high as ~5 pN s nm^−1^ (Table 1), in series with the elastic element. The underlying mechanism must rely on an activation-dependent process that, even at SL <<3 μm, excludes the contribution of the compliant tandem Ig segment. A sensible explanation is the activation-dependent formation of a link between a point in the I-band titin distal to the tandem Ig segment and the nearby actin monomer in the thin filament. The alternative explanation of an increase in the I-band titin stiffness able to attain such a high and SL-independent effective stiffness seems unrealistic, as it should interest not only the PEVK segment, the stiffness of which is Ca^2+^-sensitive (40), but also the tandem Ig segment.

### In the Activated Cell I-Band Titin Acquires Rectifying Properties Opposing Stretching and Allowing Free Shortening.

Whatever the mechanism responsible for the activation-dependent rise in the effective stiffness of the I-band titin against an increase in load, does it imply a significant resistance to muscle shortening? This question is addressed by recording the response to stepwise drops in force imposed at 3 μm SL, at which the steady passive force of ~0.1 *T*_0,c_ (Fig. 1*B*) is large enough to make the measurement feasible. In the experiment of Fig. 2*G*, apart from the response to the smallest negative force step (−9 pN, green) that maintains the multiphase aspect of the lengthening transient in response to positive steps (8 pN, magenta and 25 pN, gray), the shortening transient is characterized by a monotonic high velocity (*V*_sh_) that increases abruptly with the step size (−20 pN, orange, and −25 pN, light blue) up to values of the order of 10^3^ nm s^−1^.

Thus, a *V*–Δ*T* relation can be built for the active fiber at 3 μm SL (Fig. 2*H*), in which *V*_sh_ for negative steps, magnitude 10^3^ nm s^−1^ (triangles), is compared to *V*_3_ for positive steps, magnitude 10 nm s^−1^ (circles). The change in slope of the relation indicates that the resistance opposed by titin during active lengthening is more than two orders of magnitude larger than during shortening. Accordingly, as summarized in Table 1, I-band titin in the ON-state opposes half-sarcomere lengthening with a complex viscoelasticity characterized by a viscous element with a drag coefficient (η_3_) ~5 pN s nm^−1^, while allowing high-speed shortening as the drag coefficient (η_sh_) becomes 1.6 × 10^−3^ pN s nm^−1^ (three orders of magnitude smaller). The property of presenting a quite different resistance depending on the direction of the effort is termed rectification. This property, popular for the electric systems, in which the rectifying element is a diode, is investigated here in a mechanical system, the active half-sarcomere, in which the rectifying element is identified with the I-band titin. Changing the direction of the effort (voltage in the electric analog, force in the mechanical analog) the resistance offered by the rectifier changes so that the resulting flow (current in the electrical analog, velocity in the mechanical analog) changes by orders of magnitude. This is just how I-band titin behaves in the active half-sarcomere.

A striking feature that emerges from the analysis of the active fiber response to a negative force step is that the recoil of titin does not limit the maximum shortening velocity in control conditions. In fact, the restoring force exerted by titin at 3 μm SL (−*T*_pass_ = −24.9 ± 1.5 pN (mean ± SD), the abscissa value indicated by the arrow in Fig. 2*H*) accounts for *V*_sh_ that is more than twice the unloaded shortening velocity of myosin motors during active contraction (*V*_0_ = −2,530 ± 90 nm s^−1^ in these experiments at 4 °C, the ordinate value indicated by the arrow). Notably, *V*_sh_ is even larger if the negative force step is imposed on the resting fiber at the same SL of 3 μm (*SI Appendix*, Fig. S5). The corresponding viscosity coefficient (0.67 × 10^−3^ pN s nm^−1^) is a factor of two smaller than that of the active fiber, suggesting that the molecular interaction, hypothesized to explain the resistance to stretch of the active fiber, may affect also shortening, providing some, though much lower, viscous drag.

### An Equivalent Mechanical Model of the Half-Sarcomere Suggesting the Molecular Mechanism of I-Band Titin Switch.

**Fig. 3.**
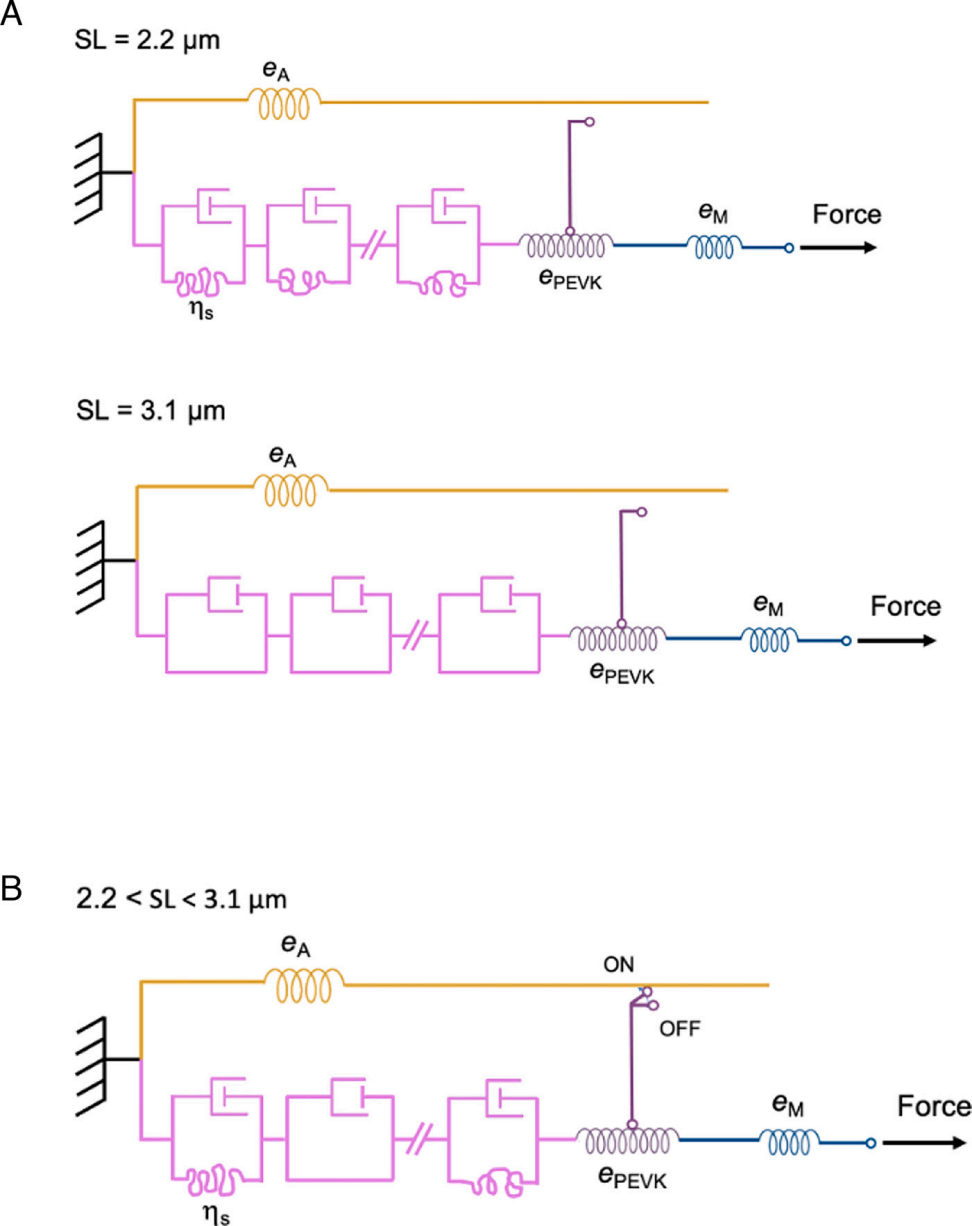
Equivalent mechanical model of the half-sarcomere describing the OFF and ON states of I-band titin in the range of SL 2.2 to 3.1 μm. Actin filament yellow, myosin filament blue, I-band titin magenta, and mechanical ground (corresponding to the Z line) black. (*A*) In the resting fiber (OFF state), the tip of the myosin filament (stiffness *e*_M_) is connected to the Z-line through the I-band titin made by two serially linked elements, the PEVK segment (dark magenta) and the tandem Ig segment (light magenta), characterized, within the limits of our analysis, by the stiffness *e*_PEVK_ and the fluidity coefficient 1/η_i_, respectively. 1/η_i_ is proportional to the number of randomly bent elements joining the Ig domains each with fluidity coefficient 1/η_s_, according to the equation 1/η_I_ = (1/η_s_)·[(*m−*1)−*k*], where (*m*−1) is the maximum number of randomly bent elements, attained at 2.2 μm SL (*Upper*), at which the fluidity is maximum and the number of straightened elements *k* is zero. *k* increases with SL and at 3.1 μm SL (*Lower*) becomes *m−*1, while the fluidity becomes zero (Fig. 2*D*), marking the SL at which the tandem Ig segment attains its contour length. (*B*) Either resting or active fiber at whatever SL between 2.2 and 3.1 μm. The hypothesized formation of the actin–titin link with the activation of the fiber is represented by the closure of the switch (from OFF to ON). The link must be distal to the tandem Ig segment to exclude its SL-dependent fluidity from the response to the force step by adding in parallel the much stiffer actin filament segment (stiffness *e*_A_) and is putatively located in the PEVK region (see text). The rectifying properties of I-band titin in the active fiber are defined by assigning to the link a viscosity coefficient η_L_ that is three orders of magnitude larger for positive force steps than for negative force steps.

Both resting and active mechanical properties of the half-sarcomere in the presence of PNB are summarized by the equivalent mechanical model of Fig. 3. The large viscous-like extensibility of the resting fiber is explained by the series of randomly bent elements joining consecutive Ig’s in the tandem Ig segment (magenta box), each resisting straightening with a viscosity coefficient η_s_, generated by the corresponding Ig movement in the solvent. At full filament overlap (2.2 μm SL, *Upper* panel in Fig. 3*A*), their number is maximum (*m*-1, for *m* consecutive Ig’s). With the increase in SL a proportionally increasing number (*k*) of segments becomes straight, with consequent decrease of the equivalent fluidity 1/η_i_. 1/η_i_ becomes 0 at 3.1 μm SL (Fig. 2*D*), at which the tandem Ig segment attains its contour length and the extensibility is accounted for by the compliance of the PEVK segment (dark magenta box, *Lower* panel in Fig. 3*A*) that is 0.65 nm pN^−1^ (Table in *SI Appendix*, Fig. S4*C*), corresponding to a stiffness of 1.5 pN nm^−1^. In the active fiber, the hypothesized formation of the link between a point in titin distal to the tandem Ig segment and the nearby actin monomer in the thin filament adds the much stiffer thin filament segment in parallel with the compliant proximal tandem Ig segment, raising the effective I-band titin stiffness to 3 pN nm^−1^ (*e*_2_, Table 1) that is the stiffness of the segment distal to the link and is independent of SL. The link is represented as a switch in the ON position and is defined by a nonlinear viscosity coefficient η_L_ with rectifying properties: with negative force steps η_L_ (=η_sh_) = 1.6 × 10^−3^ pN s nm^−1^ (Table 1), allowing free shortening, while with positive force steps η_L_ (=η_3_) = 4 to 6 pN s nm^−1^ (Table 1), resisting lengthening.

### Changes in X-ray Signals following the Force Step Reflect a Hierarchical Organization of the Structural Changes in the Myosin Filament.

**Fig. 4.**
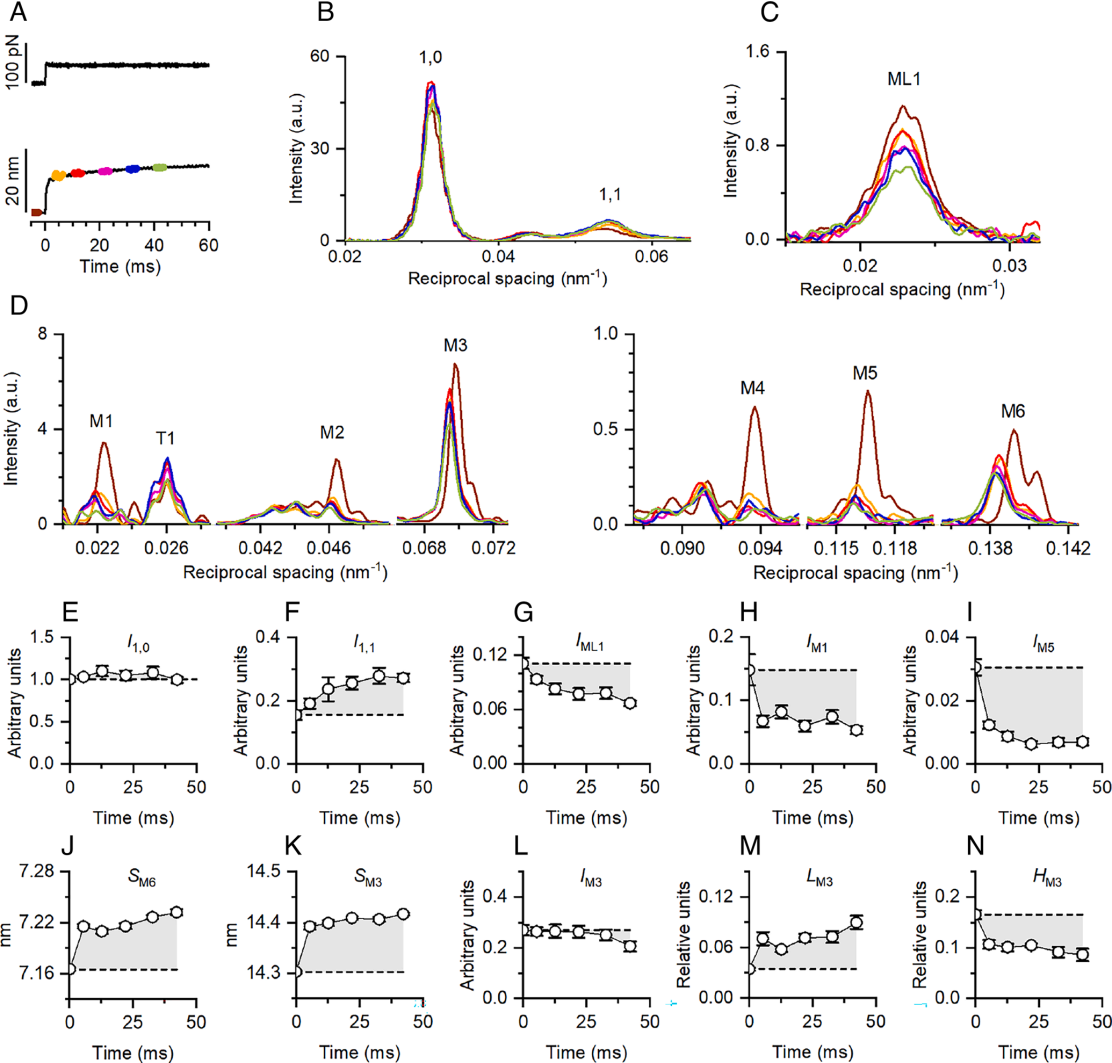
Changes in the X-ray diffraction pattern following a stepwise increase in force reflect a hierarchical organization of the structural changes in the myosin filament. (*A*) Lengthening response (per half-sarcomere, lower trace) to a force step (per htf, upper trace) of 0.22 *T*_0,c_. Colored segments denote 3 ms X-ray exposures starting at: 5 ms before the step, brown; 3, 10, 20, 30, and 40 ms after the step, orange, red, pink, blue, and green, respectively. (*B*–*D*) Intensity profiles of equatorial reflections (*B*), first myosin layer line (*C*) and meridional reflections (*D*) at times according to colors in *A*. (*E*–*I*) Intensity of the 1,0 (*E*) and 1,1 (*F*) equatorial reflections and of ML1 (*G*), M1 (*H*), and M5 (*I*). (*J* and *K*) Spacing of M6 and M3, respectively. (*L*) Intensity of M3. (*M* and *N*) Intensity ratio of the low-angle peak (*L*_M3_) and of the high-angle peak (*H*_M3_), respectively, over the total M3 intensity. In panels *E*–*N*, the horizontal dashed line is the value before the step and the shaded area marks the difference between this value and those in response to the step. Error bars are SEM. Data are from four bundles, different number of patterns (*n*) contributed to different timepoints as follows: before step, *n *= 10; after step, *n *= 9 (3 ms), 6 (10 and 30 ms), 8 (20 ms), and 10 (40 ms).

Whether the ON-state of the I-band titin provides a role for A-band titin in the mechanosensing-based activation of myosin filaments was determined using synchrotron small-angle X-ray diffraction from the muscle cell. All the X-ray signals marking the regulatory state of the filament indicate that during tetanic stimulation in the presence of 20 μM PNB, the myosin motors remain close to the surface of the filament, packed in helical tracks with 43 nm axial periodicity as at rest, notwithstanding the rise of intracellular Ca^2+^, marked by the 30% increase in the intensity of the first-order troponin-based reflection (*SI Appendix*, *Supporting Note* 2 and Fig. S3). A 50 pN force step imposed on the stimulated fiber at 2.6 μm SL (Fig. 4*A*) produces different effects, in relation to both sensitivity and timing, on the relevant reflections (Fig. 4 *B*–*N*). The intensity of the 1,0 equatorial reflection (*I*_1,0_, Fig. 4 *B* and *E*), from the lattice planes containing thick filaments, does not change significantly with the step, while the intensity of the 1,1 equatorial reflection (*I*_1,1_, Fig. 4 *B* and *F*), from the lattice planes containing thick and thin filaments, increases by 80% with a roughly exponential time-course with τ ~20 ms. The intensity of the first myosin layer line (*I*_ML1_, Fig. 4 *C* and *G*), from the three-stranded helical packing of myosin motors on the surface of the thick filament with 43 nm periodicity, decreases following the 50 pN step, attaining 60% of the intensity before the step with a timecourse similar to that of *I*_1,1_ increase. Among the myosin-based meridional reflections (Fig. 4*D*), the intensity of the so-called forbidden reflections (*I*_M1_ and *I*_M5_ in Fig. 4 *H* and *I* respectively), from the perturbation in triplets of the axial repeat of the myosin motors within the fundamental 43 nm periodicity, responds to the step with an abrupt reduction to almost ½ the value before the step. The spacing of the M6 reflection (*S*_M6_, Fig. 4*J*), reporting the extension of the thick filament, and the spacing of the M3 reflection (*S*_M3_, Fig. 4*K*), from the axial repeat of myosin motors, respond to the step with an abrupt increase by ~0.6%. The intensity of the M3 reflection (*I*_M3_, Fig. 4*L*) does not change following the step, apart a minor decrease after 30 ms, while the abrupt increase in *S*_M3_ is accompanied by similarly abrupt changes in the M3 fine structure due to X-ray interference between the two motor arrays in each thick filament (Fig. 1*A*), with ~70% increase in the intensity of the low angle subpeak (*L*_M3,_ Fig. 4*M*) and ~40% reduction in that of the high angle subpeak (*H*_M3_, Fig. 4*N*). Halving the size of the force step reduces in proportion all the changes reported above, while their timecourses following the step remain the same (*SI Appendix*, Fig. S6, triangles).

### The Stress-Dependent Myosin Filament Extension Is Synchronous with MyBP-C Link Disruption and Is followed by Azimuthal Movement of Myosin Motors.

The X-ray signals marking the regulatory state of the myosin filament show a well-defined temporal sequence of structural changes in response to the increase in load. The earliest change is the increase in filament length which is accompanied by the drop in the intensity of the forbidden reflections indicating the loss of MyBP-C–dependent triplet perturbation of the axial repeat of myosin motors. The extension of the thick filament (~2.5% per *T*_0,c_) is one order of magnitude larger than that expected from the elastic extensibility of the filament and underpins a stress-dependent structural transition reported to occur in the thick filament in the millisecond timescale (41–43). The loss of the triplet perturbation has been previously found to be associated with the reduction of overlap between actin and myosin filaments and explained with the loss of the interactions between the N-terminus of the MyBP-C and actin in the C-zone (2). Here, the loss of triplet perturbation is synchronous with the structural transition leading to the extension of the thick filament. *I*_M3_ does not change following the step and this implies that the OFF conformation of myosin motors is preserved. Both *I*_1,1_ (Fig. 4*F*) and *I*_ML1_ (G) show a slower response to the stepwise increase in force. Noteworthily, the increase in *I*_1,1_ has the same timecourse as the reduction in *I*_ML1_ and occurs without significant changes in *I*_1,0_ (Fig. 4*E*), indicating that the increase in the mass aligned with the 1,1 lattice planes, presumably constituted by myosin motors, does not imply a loss of mass aligned with the 1,0 lattice planes. This result, together with the absence of a significant reduction of the *I*_M3_ (Fig. 4*L*), poses stringent constraints for the identification of the underlying structural changes in the myosin filament.

### The Stress-Dependent Increase in Myosin Filament Extension Is Limited to the Region in which A-Band Titin Takes Periodic Interactions with Myosin Motors.

**Fig. 5.**
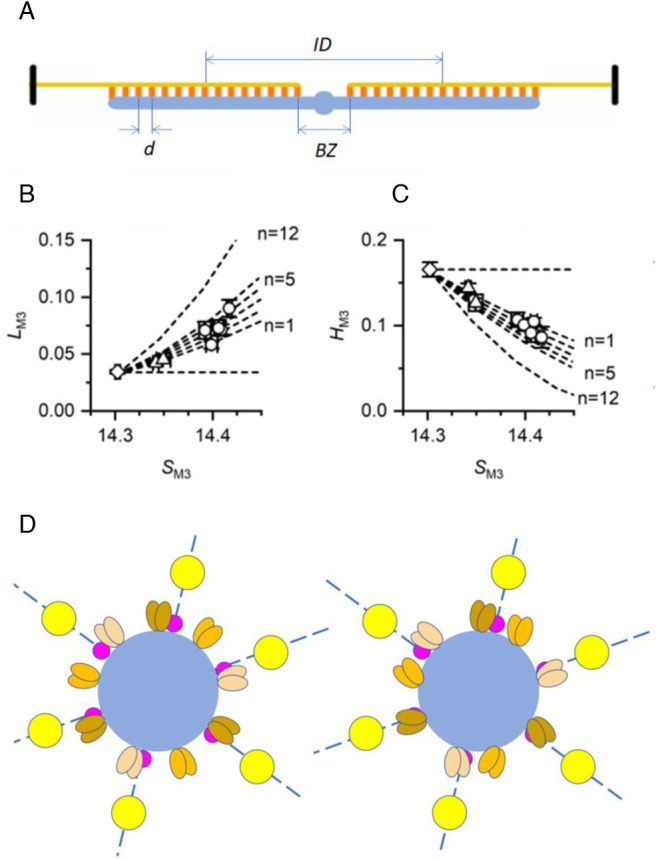
Myosin motor dispositions along and across the thick filament and their titin-related stress-sensitivity. (*A*) The two bipolar arrays of motors (orange) separated by the bare zone *BZ. d*, motor periodicity along the thick filament (blue); *ID*, interference distance. Only 15 out of 49 layers are represented for clarity. (*B*) *L*_M3_–*S*_M3_ relation using data obtained with steps of 0.11 *T*_0,c_ (triangles) and 0.22 *T*_0,c_ (circles). Diamond, data before the step. Dashed lines: simulated relations obtained assuming that the increase in *d* that explains the increase in *S*_M3_ takes places starting from the nth layer (with n = 1 for that delimiting the *BZ*) as indicated next to the line. The horizontal dashed line is obtained assuming a uniform extension, including the *BZ*, of the thick filament. (*C*) *H*_M3_–*S*_M3_ relation (symbols) and its model simulation (dashed lines) under the same conditions as detailed in *B*. In *B* and *C*, diamonds and circles are data from Fig. 4 *M* and *N*, respectively; triangles are mean data (± SEM) from the same four bundles collected at 3 ms (*n *= 8) and 40 ms (*n *= 9) after the 0.11 *T*_0,c_ step. (*D*) Schematic of the cross-section of the half-sarcomere. Blue, thick filament; yellow, thin filaments surrounding the thick filament according to the hexagonal lattice; magenta, titin disposed with a sixfold rotational symmetry on the surface of the thick filament along the same lattice planes identified by the thin filaments; and brown, orange, and ochre ovals represent the motors on three consecutive layers along the filament axis. *Left*: The three motor dimers on each layer are azimuthally separated by 120°, and each layer is rotated around the filament axis by 40° relative to the next, giving the motors a ninefold screw symmetry. *Right*: Following the force step, the stress transmitted to the thick filament and thus to the myosin–titin interactions moves motors azimuthally according to the sixfold rotational symmetry of titin.

The increase in the extension of the whole thick filament following the force step should produce an increase of the axial periodicity of myosin motors in each array (*d*, Fig. 5*A*, measured by *S*_M3_) and of the center-to-center distance between the two motor arrays in each thick filament (interference distance, *ID*, Fig. 5*A*) by the same relative amount, without phase change in the interference fringes of the M3 reflection (44). Under this condition, the structural model simulation in *SI Appendix*, Fig. S6*K* shows that the fine structure of the M3 reflection (black line) before the force step (*Left*), with *S*_M3_ = 14.3 nm, is the same as that after the force step (*Middle*), in which *S*_M3_ is increased to 14.4 nm and the same relative increase has been assigned to both the bare zone (*BZ*) and *d*. The observed changes in *L*_M3_ (Fig. 4*M*) and *H*_M3_ (Fig. 4*N*) instead indicate that the increase in *d* does not concern the whole thick filament, so that the corresponding increase in *ID* is reduced and the interference fringe pattern does not move to lower angles in parallel with *S*_M3._ The fine structure in the *Right* panel of *SI Appendix*, Fig. S6*K* is obtained by model simulation in which SM3 is increased to 14.4 nm but *BZ* and *d* of the first three layers remain the same as before the step and the increase in *d* takes place from the fourth to the 49th layer. A simulation of the *L*_M3_-*S*_M3_ and *H*_M3_-*S*_M3_ relations (Fig. 5 *B* and *C*, respectively, where circles and triangles refer to 0.22 *T*_0,c_ and 0.11 *T*_0,c_ data, respectively, and dashed lines to the simulation outputs) is developed in which different extents of the thick filament are assumed to contribute to the increase in *S*_M3_ (*SI Appendix*,  *Supporting Note* 3). Next to each dashed line, n indicates the layer (with n=1 for that delimiting the *BZ*), starting from which *d* is increased. The results of the simulation show that the observed relations are best fitted with the increase in *d* spanning the region of htf from the tip (layer 49) to layers 2 to 3 (for which R^2^ is minimized, *SI Appendix*, Fig. S6*L*). Notably, this region corresponds to that along which titin superrepeats can establish periodic interactions with the myosin motors (Fig. 1*C*).

### The Periodic Interactions of A-Band Titin with the Myosin Motors Bias their Azimuthal Orientation toward Actin.

In contrast to most of the load-dependent X-ray signals that change abruptly with the step, both *I*_1,1_ (Fig. 4*F*) and *I*_ML1_ (Fig. 4*G*) show a similar, slower timecourse indicating that the underlying structural changes are related to exponential transitions triggered by the change in load. Moreover, the increase in *I*_1,1_, which occurs without significant changes in *I*_1,0_, is just the opposite of the reduction in *I*_ML1_. A mechanism able to explain the relation between these signals is depicted in Fig. 5*D* and is based on the hypothesis that the six titin filaments (magenta) that run on the surface of the thick filament (gray) have the same sixfold rotational symmetry as the neighboring actin filaments (yellow) in the hexagonal lattice so that titin and actin lie on the same lattice planes (dashed lines). The myosin motors at rest emerge from the backbone of the filament with a ninefold screw symmetry according to the three-stranded helical packing (Fig. 5 *D*, *Left* panel; brown, orange, and ochre mark the motors on three consecutive layers), but take periodic interactions with the six titin filaments (7, 26–28). In the active fiber, an increase in load on the half-sarcomere is efficiently transmitted via the stiffened I-band titin to the thick filament, promoting some stress-dependent rearrangement in the titin–myosin interactions with a shift of the motors toward the sixfold rotational symmetry of the titin filament and thus of the actin filament (Fig. 5 *D*, *Right*). In this way, the titin–myosin interactions that drive the myosin motors with ninefold screw symmetry toward the titin sixfold rotational symmetry represent the preliminary step toward myosin–actin interaction. The finding that *I*_ML1_ and *I*_1,1_ undergo opposite changes of similar size and timecourse, while *I*_1,0_ remains constant, indicates that the azimuthal reorientation of the myosin motors occurs without any substantial change in their radial position. In this way, the mass of myosin motors associated with the thick filament does not change (*I*_1,0_ constant), while the motor mass associated with the three-stranded helix decreases (*I*_ML1_ decreases) and the motor mass aligned along the 1,1 lattice planes (dashed lines) and thus their density contrast increase (*I*_1,1_ increases).

These structural transitions depict a new mechanism of myosin filament activation, which is totally independent of the contribution of “constitutively” ON myosin motors hypothesized in previous work to trigger the mechanosensing-based thick filament activation (3). Actually, as indicated by the delayed reduction of the *I*_M3_ (Fig. 4*L*), the movement of myosin motors away from their ordered configuration on the thick filament follows their azimuthal movement from the ninefold screw to the sixfold rotational symmetry.

## Discussion

### The Activation-Dependent Changes in I-Band Titin Viscoelasticity and Its Relation to Previous Work.

The analysis of the lengthening response to a stepwise increase in force imposed on a muscle fiber in the presence of PNB reveals that the extensibility of I-band titin upon activation becomes quite small and constant independent of SL for the increase of the viscous component of the response by three orders of magnitude.

A Ca^2+^-dependent increase in the stiffness of an element in parallel with myosin motors (likely I-band titin) has been reported in previous works on intact fibers of frog and mammalian skeletal muscle (45–47) and skinned fibers and myofibrils of mammalian skeletal muscle (48, 49). In those experiments, the contribution of myosin motors was modulated by imposing rapid lengthening of different sizes (1 to 10%) on the preparation contracting under different conditions. The active nonmotor (or I-band titin) component of the force response to the stretch was measured by subtracting both the passive force response and the active isometric force developed without the stretch from the active force response to the stretch. This procedure involved two possible mistakes: i) The subtraction of the resting/relaxed force response to a stretch from the active force response is wrong if the response to the stretch of the nonmotor component changes upon activation and ii) the subtraction of the active isometric force from the active response to a stretch does not eliminate the residual unknown active response of the myosin motors to the stretch. The possible artifacts introduced with either subtraction may play in a different way at different SLs, preventing from both a reliable quantitative estimate of the stiffness of I-band titin and its SL dependence. In fact, it was found that the “nonmotor stiffness” increased with SL in contrast to the results in this work. A way to investigate the contribution of elements other than motors and myofilaments to half-sarcomere stiffness, avoiding interventions that per se change the response of the motors, has been using small 4 kHz oscillation (34) and interpreting the results with a model of the half-sarcomere comprising the I-band titin spring (50). Those experiments provided a quantitative estimate of the undamped I-band titin stiffness of ~6.5 pN/nm per htf and independent of SL (range 2.7 to 3 μm), results that are in quite good agreement with the undamped stiffness of the active fiber (*e*_1_ in Table 1) reported in the present work, in which the motor contribution is eliminated by PNB.

An original conclusion of the present work, achieved for the unique power provided by the force step protocol and the absence of myosin motors, is the finding that activation of the muscle cell increases by three orders of magnitude the viscosity coefficient of the response of the I-band titin to an increase in load, while preserving the ability to shorten freely in response to a reduction in load (Table 1). This finding accounts for the rise of the effective stiffness upon stretch to 3 pN/nm independently of SL and supports the idea that the OFF–ON titin switch is due to the interaction between titin and actin.

### Molecular Basis of the Switch of I-Band Titin Extensibility.

If the mechanism that switches I-band titin ON is its interaction with actin, it must occur at points along the I-band titin distal to the tandem Ig segment. In this way, a much stiffer element, the actin filament, is added in parallel with the tandem Ig segment (Fig. 3*B*), masking the effects of the SL-dependent extensibility of the tandem Ig segment.

A specific affinity of the PEVK segment of the I-band titin for actin is reported by several works (51–54), even if there are contradictory results on the modulatory effects of Ca^2+^ between skeletal and cardiac muscles (51, 52, 55). A direct Ca^2+^-dependent bond between the actin filament and the N2A domain of titin, which in the skeletal muscle isoform links the tandem Ig segment and the PEVK segment, has been recently hypothesized (56). The idea has been refuted based on the crystal structure of the N2A segment (57); however, it is worth noting that this structural model does not include the unique, glutamate-rich interdomain insertion of ~230 residues. On the other hand, affinity of the N2A domain for actin has been found under the control of the muscle ankyrin repeat protein MARP1, which specifically binds the N2A domain (58, 59), but in this case, titin–actin interaction accounts for a limited SL-dependent increase in passive stiffness, in contrast to the large activation-dependent and SL-independent increase in effective titin stiffness reported here. At 3 μm SL, at which the tandem Ig segment approaches its contour length, the effective I-band titin stiffness at rest is ~1.5 pN nm^−1^ (from Table in *SI Appendix*, Fig. S4*C*), ½ of that during stimulation (~3 pN nm^−1^, *e*_2_ in Table 1). Considering that the stiffness of the actin filament segment *e*_A_ is much larger than that of the PEVK *e*_PEVK_ (Fig. 3), the equivalent stiffness of the in series actin and PEVK segments must be determined by the PEVK segment, and the twofold increase in *e*_PEVK_ found here upon activation at 3 μm SL could be explained if titin attaches to actin halving the PEVK segment length distal to the link (Fig. 3*B*). Indeed, original in vitro experiments show that the PEVK poly-E motif, which has the largest actin affinity (60), is concentrated in the proximal and middle region of the PEVK segment (53) and could be the source of multiple links, as required to resist forces comparable to *T*_0,c_. However, the dependence of the actin affinity of skeletal muscle titin on ionic strength (51, 53, 54) indicates that the interaction is primarily electrostatic and whether under physiological conditions the affinity is strong enough remains to be established in future work.

The alternative mechanism of a Ca^2+^-dependent stiffening of the whole I-band titin per se appears inadequate to explain the large and SL-independent stiffness of the I-band titin of the active fiber, unless an unknown dramatic change in the properties of the tandem Ig segment was able to adapt the I-band titin contour length to match the I-band length at any SL in the physiological range.

### Titin Influences the Dynamic Equilibrium of the Interactions that Keep the Myosin Filament OFF.

The titin-dependent mechanism of myosin filament activation demonstrated here by eliminating the hampering effect of myosin motors during muscle cell activation is likely an aspect of a quite more general mechanism that controls the regulatory state of the myosin filament. The mechanism is likely based on the dynamic equilibrium between the interactions that maintain the myosin motors in the OFF conformation along the three-stranded helical tracks on the surface of the thick filament with a ninefold screw symmetry and the interactions with the A-band titin promoting their azimuthal movement toward titin sixfold rotational symmetry (Fig. 5*D*). A constitutive element of this mechanism is the increase in filament length, as shown by the increase in *S*_M6_ (Fig. 4*J*). The three-stranded helical symmetry of myosin motors in the OFF state is characterized by a fundamental axial periodicity of ~43 nm that is shared by the motor domains and MyBP-C through intramolecular (head–head and head–tail) and intermolecular (myosin–myosin and myosin–MyBP-C) interactions. The relative weakening of these interactions in favor of those with titin, which in most of the A-band is organized in C-type superrepeats (Fig. 1*C*) with fundamental axial periodicity ~45 nm [(27) and references therein], would account for a thick filament extension of the order recorded here (2.5% per *T*_0,c_).

This work gives an unprecedented quantitative and integrated description of the mechanical and structural properties of titin in the I and A bands of the sarcomere, demonstrating its multifaceted role in muscle contraction. I-band titin serves dual function through an activation-dependent switch between two states: *i*) At rest (OFF-state), titin exhibits the large extensibility permitted by the randomly oriented elements of the tandem Ig segment to adapt the half-SL to the physiological range of muscle lengths; *ii*) following activation (ON-state), the I-band titin acquires the property of a rectifier that efficiently transmits any increase in load to the thick filament without opposing free shortening at all SL. Under this condition, the periodic interactions of A-band titin with myosin motors and MyBP-C would alter the dynamic equilibrium that maintains the myosin motors in the resting conformation in a load-dependent manner. This promotes the azimuthal movement of the motors toward the 1,1 lattice planes that favors their attachment to actin.

### Significance of This Work for the Definition of Titin Functions in Health and Disease.

The quantitative description of the OFF and ON states of the I-band titin sheds light on the understanding of the efficiency of sarcomeric organization of striated (skeletal and cardiac) muscle. Macroscopic production of power during contraction of striated muscle depends on the maintenance of the ordered configuration of the half-sarcomeres over the cell cross-section for force amplification and through the serial arrangement of thousands of them for shortening velocity amplification. Differences in force among in series half-sarcomeres, combined with the descending limb of the active force–SL relation (Fig. 1*B*, black circles), would lead to lengthening of weak half-sarcomeres and rise in SL inhomogeneity. This is prevented by the high resistance to stretch of switched ON I-band titin (effective stiffness ~3 pN nm^−1^) that counter-balances the deficit in the A-band force of the weak half-sarcomeres: a deficit as high as 0.2 *T*_0,c_ (~50 pN) will be equilibrated with (50/3 =) 17 nm of I-band lengthening.

Eventually, the protocols established here open new avenues for the research on the mechanisms by which scaffold and signaling functions of titin in striated muscle are modulated by genetic, transcriptional, and posttranslational modifications related to either I-band or A-band titin (19, 61, 62). Mutations of titin gene associated with Dilated CardioMyopathy, which is characterized by systolic dysfunction with thinning and expansion of ventricular wall and accounts for up to 50% of cases of heart failure (63, 64), lead to titin truncation in either its I-band or A-band domains. The common resulting phenotype is the deficit in contractile force, but the underlying molecular mechanisms are quite different and can be envisaged thanks to the new integrated description of I-band and A-band titin functions in this work. Truncations in the I-band titin may affect its switching ON blunting the force equilibration among in series half-sarcomeres that prevents sarcomere disarray during contraction, while truncation in the A-band titin may affect contractility by blunting the titin-based switching ON of myosin motors.

The methodological approach and the concepts established in this work represent powerful new tools for investigations in demembranated muscle fibers from wild-type, mutant, and engineered mammalian models and from human biopsies aimed at defining the genotype–phenotype relation at the level of the basic function of a titin variant and developing the specific therapeutical strategies.

## Materials and Methods

The experiments were made on single muscle fibers or small fiber bundles (2 to 3 fibers each) isolated by dissection from the skeletal muscle (tibialis anterior or lumbricalis) of *Rana esculenta*, the preparation that uniquely allows the passive resistance to stretch to be attributed solely to an intracellular elastic component like titin (10, 11). Frogs were killed in agreement with the Authorization 956/2015-Progetto di Ricerca from the Italian Government and the European Union directive 2010/63. Mechanical experiments have been carried out at the PhysioLab, Department of Biology, University of Florence, Florence, Italy. Combined X-ray diffraction and mechanical experiments have been carried out at the beamline ID02 of the European Synchrotron Radiation Facility (ESRF), Grenoble, France (65). Biochemical assays on the actin-activated ATPase activity of HMM and S1 fragments of frog myosin, extracted at the PhysioLab from the leg muscles of frogs belonging to the same batch of the frogs used for mechanical experiments, were done at the Department of Biochemistry, Eötvös University, Budapest, Hungary.

For the mechanical experiments, the fiber was horizontally mounted in a thermoregulated, anodized aluminum trough between the lever arms of a capacitance force transducer (66) and a loudspeaker motor (range of movement ± 600 μm, upgraded from the original design of ref. 67) by means of aluminum clips. The top of the trough was covered with a cover-glass carrying two platinum plate stimulating electrodes running parallel to the fiber. A striation follower (68) was used to record the length changes of a population of ~500 sarcomeres selected in the third of the fiber near the force transducer end (*SI Appendix*, *Materials and Methods*). During the experiment, the temperature in the trough was continuously monitored and maintained constant at 4 °C. Tetanic contractions were elicited with a train of even number of stimuli of alternate polarity (stimulation frequency 18 to 25 Hz) applied transversely to the muscle fiber by means of the platinum electrodes. In PNB experiments, before starting the perfusion with PNB, the fiber bundle was tetanically stimulated in control Ringer solution and 2.15 μm SL in order to record the reference tetanic force *T*_0,c_. Following complete inhibition of the isometric force development upon stimulation in PNB solution, stepwise increases in force of different amplitude (0.1 to 0.4 *T*_0,c_) were imposed, after switching the control of the loudspeaker-motor servo-system to force feedback, both at rest and at 60 ms following the start of tetanic stimulation to elicit the isotonic lengthening transient at different SLs (2.3, 2.5, 2.7, and 3.0 μm). For the X-ray measurements, the trough was adapted to allow minimization of the X-ray path through the solution and sealed for vertical mounting of the fiber at the beamline. The load-dependence of the X-ray signals in PNB Ringer was determined in the stimulated fiber at 2.6 to 2.7 μm SL by imposing force steps of 0.11 and 0.22 *T*_0,c_ 60 ms after the start of stimulation and recording 2D patterns with 3 to 5 ms time windows 5 ms before the step and at different times following the step. Details on the mechanical and X-ray diffraction protocols and analyses and on the biochemical assays are provided in *SI Appendix*, *Materials and Methods*.

### Statistical Analysis.

Data are expressed as mean ± SD or SEM as specified. The number of fibers contributing to each protocol is reported in the text and in the figure and table legends. Statistical significance was determined using the two-tailed *t *test, assuming the level of significance *P *< 0.02.

## Supplementary Material

Appendix 01 (PDF)

## Data Availability

All relevant data, associated protocols, and materials are within the paper and/or *SI Appendix*. Raw data from X-ray experiments are available at https://doi.org/10.15151/ESRF-ES-517789918. Raw data from mechanical and biochemical experiments are available at https://zenodo.org/record/7614544#.Y-NcOXbMK5d.
