# Supplementary material for: Titin activates myosin filaments in skeletal muscle by switching from an extensible spring to a mechanical rectifier
Source: Proc Natl Acad Sci U S A. 2023 Feb 22;120(9):e2219346120. doi: 10.1073/pnas.2219346120 (PMC9992839; doi:10.1073/pnas.2219346120)
Supplement: Supplementary file 1 — Appendix 01 (PDF) [file pnas.2219346120.sapp.pdf]

PNAS

Supporting Information for

Titin activates myosin filaments in skeletal muscle by switching from an extensible spring to a mechanical rectifier

Caterina Squarci, Pasquale Bianco, Massimo Reconditi, Irene Pertici, Marco Caremani, Theyencheri Narayanan, Ádám I. Horváth, András Málnási-Csizmadia, Marco Linari, Vincenzo Lombardi* and Gabriella Piazzesi

***Corresponding author:** Vincenzo Lombardi
vincenzo.lombardi@unifi.it

This PDF file includes:

Supporting text:

Supporting Materials and Methods

Supporting Notes 1 to 3

Figures
S1 to S6

SI References

Supporting Information Text

Supporting Materials and Methods

Experimental model and subject details. The experiments were done on single muscle fibers or small fiber bundles (2-3 fibers each) isolated from the tibialis anterior or lumbricalis muscles of *Rana esculenta*. Frogs (ca. 50 g weight) were killed by percussive blow to the head followed by the destruction of the spinal cord in agreement with the Authorization 956/2015-PR from the Italian Health Ministry in compliance with Decreto Legislativo 26/2014 and with EU directive 2010/63. The intact fiber isolated from frog muscle is the best preparation for these experiments because in the frog muscle fiber (*i*) the passive resistance to stretch is solely due to titin (1, 2) and (*ii*) sarcomere level mechanical measurements by means of a striation follower (3) allow to minimize inertial effects (4). Mechanical experiments have been carried out on fibers isolated from the tibialis anterior muscle at the PhysioLab, Department of Biology, University of Florence, Florence, Italy. This muscle is preferred to other hind-limb muscles for the quality of dissection and fiber length (l_0, the fiber resting length, that is the length at 2.15 μm sarcomere length (SL), is ~5 mm), factors that are relevant for the optimization of fast sarcomere level mechanics. Combined small-angle X-ray diffraction and mechanical experiments have been carried out on fiber bundles from both the tibialis anterior and lumbricalis muscles, at the beamline ID02 of the European Synchrotron (ESRF), Grenoble, France (5). The experiments to measure the changes in X-ray signals at various times following a force step have been done only on tibialis anterior fiber bundles. The use of bundles of two-three fibers for X-ray diffraction experiments is preferred for a diffracting mass large enough to have a signal-to-noise ratio adequate to measure the weaker reflections. The biochemical assays on the actin-activated ATPase activity of HMM and S1 fragments of frog myosin, extracted at the PhysioLab from the leg muscles of frogs belonging to the same batch of frogs used for mechanical experiments, were done at the Department of Biochemistry, Eötvös University, Budapest, Hungary.

Method details. *Preparation and mounting of the fibers for mechanical and X-ray diffraction experiments.* Fiber dissection was performed under a stereomicroscope (Stemi SV6 or SteREO Discovery V8, Zeiss) with the aid of small knives, tweezers and scissors, at room temperature. To minimize the amount of tendon compliance in series with the sarcomeres, the tendons were clipped to a length as short as possible (length at either end ~150 μm) with aluminum foil clips (6). Special care was taken in dissecting and mounting the fiber in order to avoid transverse movements during contraction or imposed length/force changes.

For mechanical experiments, the fiber was horizontally mounted in a thermoregulated trough between the levers of a capacitance force transducer (7) and a loudspeaker motor (range of movement ± 600 μm, upgraded from the original design described in (8)) by means of the aluminum clips. During the experiment, the temperature in the trough was maintained constant at 4 °C and the fiber was stimulated by means of two platinum plate electrodes running parallel to its axis. The whole system (experimental trough, loudspeaker motor, force transducer) was carried on a metal plate connected to the movable stage of a microscope stand (ACM, Zeiss).

For combined X-ray diffraction and mechanical experiments, the fiber (or the fiber bundle) was mounted in a thermoregulated trough adapted for X-ray measurements. Two hollow cylinders, carrying two mica windows and the stimulating electrodes, were moved as close as possible to the fiber, to minimize the X-ray path through the solution. The gap between the windows was typically 600 μm. To have the fiber axis parallel to the smaller (vertical) size of the X-ray beam and maximize the spatial resolution of X-ray signals along the meridional axis,

parallel to the fiber axis, the trough was sealed and mounted vertical at the beamline, with the force transducer on the top and the motor at the bottom.

Measurements and stimulation of the fiber. Sarcomere length (SL), fiber length, height (*h*) and width (*w*) were measured under ordinary light on the movable stage of the ACM microscope using a 40x water immersion Zeiss objective and a 25x eyepiece. Fiber length was initially adjusted to have an average SL of 2.15 µm. Different SL (range 2.3-3 µm) were obtained by increasing the fiber length until the desired SL was attained as controlled under the microscope. Cross sectional area (CSA) was calculated from *h* and *w*, as if they were the axes of an elliptical section.

Tetanic contractions were elicited with a train of even number of stimuli of alternate polarity applied transversely to the muscle fiber by means of the platinum electrodes across which up to 10 V could be applied with a constant-voltage pulse generator. Stimuli of 1.5 times the threshold and 0.5 ms duration were used. The optimal stimulation frequency, the minimum frequency for a fused tetanus at 4 °C, ranged 18-25 Hz and was kept for the subsequent phase of the experiment in the presence of the myosin inhibitor PNB.

Solutions. The physiological solution (Ringer) had the following composition: 115 mM NaCl, 2.5 mM KCl, 1.8 mM $CaCl_2$, 3 mM phosphate buffer at pH 7.1. Para-nitro-blebbistatin, PNB, was dissolved in dimethyl sulfoxide (DMSO, PNB stock solution concentration 13 mM) and added to the Ringer solution to have a final PNB concentration of 20 µM. The addition of DMSO alone to control Ringer at the final concentration (22 mM) as that in the PNB Ringer did not alter the mechanical response of the fiber.

Biochemical assay. The preliminary comparative analysis of the inhibitory action of blebbistatin and PNB (9) was conducted at the Department of Biochemistry, Eötvös University (Hungary), titrating the effects of the two inhibitors on the actin-activated ATPase activity of S1 and HMM fragments of myosin extracted from the frog leg muscles at the PhysioLab, University of Florence. As shown in Fig. S2A and B, 20 µM PNB completely block the actin-activated ATPase activity of both frog myosin fragments, while in 20 µM blebbistatin significant residual ATPase activity can be detected (1.1 ± 0.2% and 4.9 ± 0 .5% for S1 and HMM, respectively). Steady-state actin-activated ATPase activity measurements were carried out in 50 µL volume in a flat bottom 384-well plate (Nunc-Thermo Fischer) using NADH-PK/LDH coupled assay described previously (10). Briefly, frog muscle HMM or myosin-S1 samples were mixed with 2% pyruvate kinase/lactate dehydrogenase (PK/LDH) mixture (Sigma P0294), 1 mM phosphoenol-pyruvate (PEP) and 200 µM NADH at 25 °C in the presence of 0.5 mM ATP and 25 µM F-actin in ATPase buffer (10 mM MOPS pH 7.0, 4 mM $MgCl_2$, 2 mM b-mercaptoethanol) for 15 minutes. Absorbance at 340 nm wavelength was recorded to follow the decrease of NADH in Microplate Spectrophotometer (BioTek Epoch). ATPase activity was calculated from linear regression of the time dependent absorbance data collected at 340 nm. Different concentrations of inhibitors were added to the reaction in 0.5 µL DMSO (1% of total volume). DMSO and actin-controls were measured for each measurement set.

Mechanical experiments. *Mechanical apparatus.* The force was recorded by means of a capacitance gauge transducer (resonant frequency 30 - 50 kHz, sensitivity 80 - 150 mV/mN and noise 2 - 8 mV peak-to-peak) similar to that described in (7). A striation follower (similar to that described in (3)) was used to record the length changes of a population of ~500 sarcomeres selected in the third of the fiber near the force transducer end. Systematic errors that could result from inhomogeneity of the sarcomere length within the population and from changes of the sarcomere length upon activation were minimized by using fibers in which sarcomere inhomogeneity at rest was less than 5% and discarding fibers developing gross inhomogeneities on activation.

The loudspeaker motor was servo-controlled using as the feedback signal the output from either the position sensor on the motor lever (motor position clamp, P_m-clamp mode) or the force transducer (force-clamp mode). To impose force steps on the fiber, the motor was first operated in P_m-clamp mode and then, at a pre-set time before the force step, was switched to force-clamp mode by a command signal. The return to P_m-clamp mode was operated either at a pre-set time by a second command signal or whenever the signal from the loudspeaker position sensor exceeded the values set in a couple of comparators. This procedure provided that the length change required to maintain the force imposed on the fiber could not exceed the range of movement (± 600 μm) within which the motor-length transducer behaves linearly and safely.
The step perturbation in force-clamp mode is the most powerful protocol to investigate the structural dynamics of molecular and intermolecular processes because, following the step, the force is kept constant so that the length response of the sarcomeres is not influenced by any length change of in-series compliance and records the molecular transformation under a constant potential energy landscape (11, 12). The main limit to the effectiveness of the method is related to the inertia of the fiber that introduces a delay in the feed-back loop limiting the gain of the system and thus the frequency domain of the force step. In these experiments, inertial effects on the measurements were minimized by exploiting the striation follower to record half-sarcomere length changes near the force transducer end (4).
Mechanical protocols. At the start of the experiment isometric tetani were elicited under P_m-clamp mode in normal Ringer solution (control solution, 4 °C) at 4 min intervals with trains of stimuli of duration ~300 ms at 2.15 μm SL to establish the reference value of the isometric plateau force ($T_{0,c}$). The unloaded shortening velocity (V_0) of the stimulated fiber was measured from the selected population of sarcomeres by imposing, in P_m-clamp mode, a steady shortening of size and velocity sufficiently large to drop/keep the force to zero. Then the temperature was set to 14 °C and the fiber perfused with Ringer solution containing 20 μM PNB, the concentration adequate to attain 100% inhibition of the *in vitro* steady-state actin-activated ATPase activity (Fig. S2A and S2B) and of force elicited by tetanic stimulation (Fig. S2C). The fiber was stimulated every ~5 minutes to control the progression of the effect of PNB on force, which takes ~50-100 minutes to be complete (Fig. S2D and S2E). The temperature was then set again to 4 °C. Stepwise rises in force (positive force steps) of amplitude ΔT ranging 0.1-0.4 $T_{0,c}$ were imposed in force-clamp mode both at rest and at 60 ms following the first stimulus during tetanic stimulation to elicit the isotonic lengthening transient at different sarcomere lengths (2.3, 2.5, 2.7 and 3.0 μm). 4 minutes intervals were interposed between subsequent records. The size of the force step and the sarcomere length were set in a random sequence. In 8 of the 22 fibers used in this work, the shortening transient in response to a stepwise drop in force (negative force steps) was determined at different times during stimulation at starting SL 3.0 μm, at which a restoring force of ~0.1 $T_{0,c}$ (or ~25 pN per half-thick filament) is present at rest (Fig. 1B). In 5 of these 8 fibers the negative force steps were imposed also at rest.
X-ray Diffraction Experiments. Data in control and in PNB Ringer solution cannot be reliably collected from the same fiber bundle because, given the long time for full force inhibition by PNB, the radiation damage on a formerly exposed sample could have progressed so as to affect the PNB responses. Thus, control and PNB experiments were in general carried on different preparations. The beam size at the detector was ~150 μm x 30 μm (horizontal x vertical, Full Width at Half Maximum, FWHM) with a flux 10^{13} photons/s at a wavelength of ~0.1 nm (5) that was attenuated for bundle alignment. To minimize radiation damage the trough was

vertically shifted by 100-200 μm between X-ray exposures and two fast electromagnetic shutters in series (tandem shutters, (5)) were used to shape the exposure windows and limit the X-ray exposure times to the data collection windows.

X-ray diffraction protocols. In control experiments, 2D diffraction patterns were first collected with 5 ms time windows at rest and at the plateau of the isometric tetanus at 4 °C and SL 2.15 μm. At rest, the bundle was slowly stretched to 2.6-2.7 μm SL and the protocol repeated (Fig. S3). A SL ≤2.7 μm was chosen because this is the largest SL at which X-ray data can be collected with minimal effects of radiation damage (13).

In PNB experiments, before starting the perfusion with PNB, the fiber bundle was tetanically stimulated in control Ringer solution at 4 °C and 2.15 μm SL in order to record the control tetanic force $T_{0,c}$. The bundle was then perfused with PNB Ringer, the trough was sealed and mounted in the path of the X-ray beam. Following complete inhibition of the isometric force development upon stimulation, 2D diffraction patterns were collected at 2.15 and 2.6-2.7 μm SL, at 4 °C, with 5 ms time frames at rest and during tetanic stimulation at the same frequency as that used to record the control tetanus at 2.15 μm SL (Fig. S3). The load-dependence of the X-ray signals in PNB Ringer was measured imposing force steps of 0.11 and 0.22 $T_{0,c}$ on the stimulated fiber bundle 60 ms after the start of stimulation and 2D patterns in 3-5 ms time windows were collected 5 ms before the step and at different times following the steps: 3 and 40 ms for 0.11 $T_{0,c}$ and 3, 10, 20, 30 and 40 ms for 0.22 $T_{0,c}$ (Fig. 4 and S6).

Mechanical data collection and analysis. Force, motor position and half-sarcomere length changes were recorded with a multifunction I/O board (PXIE-6358, National Instruments). A program written in LabVIEW (National Instrument) was used for signal generation and data acquisition. Data analysis was performed using Excel (Microsoft), OriginPro 8.0 (OriginLab Corporation) and programs written in LabVIEW.

The lengthening (L_2) attained at the end of the rapid phase 2 of the transient in response to a positive force step (Fig. 2A, left panel) was estimated by extrapolating back to the half-time of the step (vertical line) the tangent (black line) to the later part (phase 3) of the transient. V_3, the steady velocity of the phase 3 lengthening was estimated by the slope of the tangent. In the stimulated fiber an elastic phase 1 response simultaneous with the force step is clearly distinguishable from phase 2 (Fig. 2A, right panel, light green trace). L_1, the amplitude of phase 1 lengthening, was estimated by extrapolating back to the half-time of the step the tangent to the initial part of the phase 2 lengthening. In the resting fiber there is no evidence for an elastic phase 1 response simultaneous with the step. Instead, the initial lengthening velocity V_i can be estimated as the slope of the tangent to the initial 0.5 ms of the length trace following the step end (Fig. 2A, right panel dark green trace). The response to negative force steps imposed on the active fiber at 3 μm SL, apart for the very small steps, is characterized by a monotonic shortening at high velocity V_{sh} (Fig. 2G, orange and light blue traces). A monotonic shortening at a much higher V_{sh} is the only response to negative force steps of the same amplitudes imposed on the resting fibers (Fig. S4).

Force (pN) per half thick filament (htf) has been calculated from force per fiber CSA, with a density of thick filaments of $5.87 \cdot 10^{14}$ m^{-2} (14,15). The L_2-ΔT and V_3-ΔT relations for positive steps were fitted with linear regressions to data pooled from the 22 fibers using a built-in function of OriginPro software. Given the large and dispersed values of V_{sh} in response to a quite narrow range of negative force steps (see for instance triangles in Fig. 2H), the V_{sh}-ΔT relation was fitted individually for each fiber and then the estimates of the slopes and intercepts were averaged.

X-ray data collection and analysis. 2D diffraction patterns were collected on a FReLoN (Fast Readout Low-Noise) CCD (Charge Coupled Device) detector with active area 50×50 mm^2, 2048×2048 pixels (binned by 8 in the horizontal direction before the read out to increase the signal-to-noise ratio) and point spread function (PSF) about 44 μm (FWHM) (5). Tandem shutter opening and CCD data acquisition were synchronized with the timing of the mechanical protocols by using the same LabVIEW program as that used for mechanical experiments. The intensity of the beam incident on the sample and the time windows of exposures were recorded by a reference pin diode.

The 2D X-ray patterns were corrected on-line for dark current, flat field response and spatial distortion of the detector. The off-line analysis of X-ray data was performed in the laboratory in Florence using Fit2D (Hammersely, ESRF), PeakFit (SeaSolve Software Inc.), IgorPro (WaveMetrix Inc.) and Origin software. Single 2D patterns were shifted and rotated to have the four quadrants symmetric relative to the center of the image using the equatorial 1,0 reflection position, then quadrant folded (mirrored) to enhance the S/N ratio.

The 2D patterns (Fig. S3A) were integrated along the meridian or equatorial axis to get 1D intensity profiles. The distribution of diffracted intensity along the meridional axis was determined by integrating 0.012 nm^{-1} on either side of the meridian for including and measuring the whole intensity of the reflections and 0.0046 nm^{-1} for measuring their spacings and fine structure. For ML1 layer line, the intensity distribution in the direction parallel to the meridional axis was obtained by radial integration between 0.064 and 0.037 nm^{-1} from the meridional axis. The distribution of diffracted intensity along the equatorial axis was obtained by integrating 0.0036 nm^{-1} on either side of the equator.

The background intensity distribution was determined using a convex hull algorithm and subtracted (Fig. 4B-D and S3C-E). The 1D intensity profiles of the reflections (except for the forbidden reflections and ML1) were fitted with a Gaussian peak (1,0 and 1,1, fit limits 0.029-0.041 nm^{-1} and 0.052-0.070 nm^{-1}, respectively) or multiple Gaussian peaks with the same axial width (M3 and M6, fit limits 0.067-0.072 nm^{-1} and 0.133-0.144 nm^{-1}, respectively). The total intensity of a reflection was calculated as the sum of the component peaks and its spacing was determined from the intensity-weighted mean of the centers of the component peaks, calibrated using as a reference the position of the M3 reflection in the fiber at rest at full overlap (sarcomere length 2.1-2.2 μm), taken as 14.34 nm (16). The intensities of M1 and M5 reflections were measured by integrating their 1D distributions along the meridian between 0.020 and 0.025 nm^{-1} and between 0.114 and 0.119 nm^{-1} respectively. The intensity of ML1 reflection was obtained by integrating its 1D distribution parallel to the meridional axis between 0.019–0.023 nm^{-1} corresponding to the half, low angle side of the reflection to avoid contamination with the overlapping, non-resolved AL1 layer line from the actin helix (17).

The observed intensity of the reflections increases with the number of myofilaments in the X-ray beam, which depends on the CSA of the fiber bundle and varies with inverse proportionality to the sarcomere length. Also, it could be modulated by possible inhomogeneities along the bundle. To correct for these factors and make the results from different fiber bundles consistent, the intensities of the reflections were scaled by the intensity of the equatorial 1,0 reflection ($I_{1,0}$) at rest as measured on the same pattern at the same SL. The validity of this normalization procedure relies on the finding that $I_{1,0}$ at rest in different fibers depends solely on the number of myofilaments in the X-ray beam as demonstrated by $I_{1,0}$ remaining intrinsically constant once corrected for the fiber mass under the beam (13).

***Quantification and statistical analysis*.** Data are expressed as mean ± SD or SEM as specified. Mechanical experiments were done on 22 fibers dissected from as many frogs. Given the complexity of the experimental

design (combination of force steps of different sizes at different SL) not all the fibers contributed to all the protocols. The number of fibers contributing to each protocol is reported on the text and in the Figure and Table legends. The values of *n* reported for a given parameter refer to the number of repeats contributing to estimate the mean of that parameter.

The total number of preparations used for X-ray experiments was twelve from as many frogs. Four bundles were used for measurements in control solution and eight for determining the effects of PNB (four lumbricalis and four tibialis anterior). Statistical significance was determined using two-tailed *t*-test, assuming the level of significance $P < 0.02$.

Supporting Note 1

The molecular basis of passive force-SL relation

In this work we assume that the passive force-SL relation of the single fiber from frog skeletal muscle can be explained by the contribution of two spring elements with different extensibility, serially linked in the I-band titin (18-20): (*i*) the proximal tandem Ig segment that in the SL range 2-2.7 μm behaves as an entropic spring with a large persistence length (L_p) and thus lengthens with development of very low force, and (*ii*) the PEVK segment that, at SL ≥ 2.8 μm, at which the tandem Ig spring approaches its contour length (L_c) becoming inextensible, responds to further stretch with rise in force according to a much shorter L_p. In the absence of direct information on the molecular structure of the frog titin, this explanation of the frog passive force-SL relation relies on the definition of the contributions of the two spring elements in the rat psoas myofibril (19, 21). Justifications for this assumption are that (*i*) in either preparation the passive force-SL relation is free from the contribution of the extracellular matrix (2), and (*ii*) the titin structure is mostly conserved in ortholog isoforms of the fast skeletal muscle of vertebrates. The assumption is tested here by conducting a comparative analysis of the force-SL relation of the two experimental models (Fig. S1, blue dashed line rat psoas myofibril from (21)), red dashed line frog muscle fiber from Fig. 1B). These two preparations share the same myosin filament length (l_M = 1.6 μm) and therefore the force-SL relation can be uniquely expressed also as force versus the I-band titin length (l_T = (SL – l_M)/2; lower abscissa). The two relations exhibit a similar large extensibility up to SL ~2.8 μm and then diverge, with the frog fiber relation rising more steeply. This qualitative comparison suggests that at SL > 3 μm, at which the PEVK contribution to extensibility becomes dominant, the frog relation exhibits a larger steepness. For a quantitative test we used the I-band titin model that implies the sequential extension of two serially linked WLC's (18-23): the first WLC, constituted by the tandem Ig segment, with a purely entropic elasticity and the second WLC, constituted by the PEVK segment with entropic and enthalpic elasticities. We found that fitting the psoas myofibril relation (blue circles in Fig. S1) gives almost the same estimates of the relevant parameters of the model as in the original works (Table in Fig. S1), indicating the reliability of our simulation procedure. Model fitting of the frog fiber relation (red circles in Fig. S1, reported also in Fig. 1B) gives L_p values of the tandem Ig segment and of the PEVK segment slightly larger than those of psoas myofibril, accounting for the frog relation lying below the psoas relation in the range 2.4-2.9 μm SL. The Young modulus *E*, characterizing the enthalpic contribution, was 80% larger in the frog fiber (Table in Fig. S1) accounting for the larger steepness of the force-SL relation at SL>2.9 μm. The comparative analysis indicates that in either preparation the tandem-Ig segment attains its contour length at an I-band titin length (660-680 nm) that corresponds to ~3 μm SL.

Supporting Note 2

Structural evidence for 100% inhibition of myosin filament activation by PNB

Addition of 20 μM PNB to Ringer solution induces, within 50-100 min, the complete suppression of the force generated by single fibers or bundles of 2-3 fibers of the hind-limb skeletal muscle of the frog under tetanic stimulation (Fig. S2C-E and Fig. S3B, blue normal Ringer solution, orange in the presence of PNB). The structural correlate of the action of PNB at the level of the thick filament and myosin motors was determined by collecting 2D small-angle X-ray diffraction patterns at the ID02 beamline of the European Synchrotron (Grenoble, France). For the purpose of this work, low angle X-ray diffraction patterns were collected at SL 2.6-2.7 μm (see Supporting Materials and Methods).

A typical 2D pattern at rest is shown in Fig. S3A. The reflections along the horizontal axis, orthogonal to the fiber axis, are called equatorial reflections and are due to the crystallographic planes originating from the regular disposition of the myofilaments in the lattice. Among them, the most intense are the so-called 1,0 (associated with the lattice planes containing thick filaments) and 1,1 (associated with the lattice planes containing both thick and thin filaments). The ratio of the intensities of the 1,1 and 1,0 reflections ($I_{1,1}/I_{1,0}$) is considered a signal for the activation-dependent movement of the myosin motors away from the thick filament towards the thin filament (24). The off-meridional layer lines parallel to the equatorial axis originate from structures with helicoidal symmetry along the filaments. Among them, the first myosin layer line reflection at ~43 nm (ML1), is due to the three-stranded helical packing of the myosin motors on the surface of the thick filament (Fig. 1C, inset) (25). The vertical axis, parallel to the fiber axis, is called the meridian and the reflections along it are called meridional reflections. Those indexed M1 to M6 are orders of a fundamental axial periodicity of ca. 43 nm associated with myosin and are split in closely spaced subpeaks arising from X-ray interference between the two arrays of myosin motors in each thick filament (Fig. 5A, see also (26)). The T1 reflection, next to the high angle side of the M1 (Fig. S3E), originates from the axial ~38 nm periodicity of troponin on the thin filament (Fig. 1C, inset). Among the myosin-based meridional reflections, the strong M3 originates from the axial repeat of myosin motors with periodicity 14.34 nm (16), and the M6 originates mainly from a periodic mass distribution in the thick filament backbone with periodicity 7.17 nm (27, 28). The presence of M1 and the so-called "forbidden" reflections M2, M4 and M5 indicates that, in each 43 nm axial period, the mass distribution of myosin heads is not composed of identical 14.34 nm repeats as expected from a perfect three-stranded helix, likely because of the contribution of the MyBP-C, which is present in the C-zone of the thick filament (Fig. 1C, green) with a 43 nm periodicity (29-33). Given the much smaller mass, MyBP-C cannot directly contribute to the forbidden reflections but can just perturb myosin axial periodicity through its interaction with both the myosin, within the 43 nm periodicity (30), and the nearby actin filaments, by means of its N-terminal domain (29, 34, 35), as proven by the linear drop of the intensity of the forbidden reflections in the SL range (2.5 – 3.1 μm), in which the overlap between thick filament C-zone and thin filament progressively reduces from full to zero (Fig. 1C-D; (13)).

In Fig. S3C-I, X-ray signals collected at 2.7 μm SL in normal Ringer solution (black at rest, blue at the plateau of the isometric tetanus, force T_0) are compared with those in the presence of PNB (brown at rest, orange during tetanic stimulation). Tetanic stimulation in control produces (black to blue): (*i*) increase of the intensity ratio of the low angle equatorial reflections ($I_{1,1}/I_{1,0}$) from the resting value of 0.16 to the T_0 value of 0.58 (Fig.

S3C and S3F), indicating movement of the myosin motors from the surface of the thick filament toward the thin filament; (*ii*) reduction to ¼ of the intensity of the ML1 layer line (I_{ML1}) (Fig. S3D and S3G), indicating that the helical symmetry of the motors on the surface of the thick filament reduces and the motors are either disordered or attached to actin; (*iii*) reduction of the intensity of the meridional reflections M1-M6, indexing on the axial periodicity of ~ 43 nm (Fig. S3E), with the exception of M3 and M6 that remain strong; (*iv*) change of M3 fine structure from a main peak at 14.35 nm and small satellite peaks on either side (black) to two peaks of similar intensities (blue). The ratio of the high angle peak intensity over M3 total intensity (H_{M3}) is 0.15 at rest and becomes 0.50 at T_0 (Fig. S3E and S3H), indicating that myosin motors have moved from their resting configuration, in which they lie on the surface of the thick filament tilted back on their tails in the OFF state (inset in Fig. 1C; (36, 37)), to either disordered or attached to actin with the lever arm tilted near the perpendicular to the filament axis (36, 38-40); (*v*) increase of the spacing of M6 reflection (S_{M6}, Fig. S3E and S3I) from 7.176 nm (black) to 7.271 nm (blue), 5 times larger than the increase expected from the elasticity of the filament (28, 41) and correlated with slower stress-induced structural changes in the thick filament (42-44). The perfusion with PNB Ringer solution does not significantly affect any of the X-ray signals at rest (compare brown and black in Fig. S3C-I). Upon tetanic stimulation in the presence of PNB all the myosin-based X-ray signals maintain the values characteristic of the resting state (compare brown and orange in Fig. S3C-I), apart a significant increase in the intensity of the 1st order troponin-based reflection (I_{T1}, +32 ± 9%, P <0.005).

In this respect it must be noted that I_{T1} is known to increase early during muscle stimulation in control at full filament overlap, attaining a peak ~1.3 the resting value at the end of latency relaxation, and then monotonically decrease during the force development up to a value less than 0.2 the resting value (36, Fig 4A; see also 46). The early I_{T1} increase was attributed to the Ca^{2+}-dependent structural change in troponin-tropomyosin (45, 46), while the later reduction was associated with motor attachment to the actin filament. This conclusion is supported also by the finding that the early increase in I_{T1} to ~1.3 the resting value is preserved when the fiber is stimulated at SL beyond filament overlap at which force development is suppressed (46). Accordingly, here we find that, in the presence of PNB which prevents myosin motor attachment, I_{T1} increases by ~30% upon stimulation. This finding in turn is a check that PNB per se does not affect increase in intracellular Ca^{2+} and thin filament activation upon stimulation.

Previous work that used skinned mammalian fibers and blebbistatin (100 μM) failed to attain, upon Ca^{2+} activation, full force inhibition and full preservation of the relaxed structure (47,48). Instead, using intact frog fibers and PNB, we show that tetanic stimulation does not elicit any mechanical and X-ray structural response by myosin motors, indicating that their resting configuration, in which they lie on the surface of the thick filament tilted back on their tails in the OFF state is fully preserved. As demonstrated by the comparison of the inhibition of the actin-activated ATP-ase of both S1 (Fig. S2A) and HMM (Fig. S2B) fragments of frog myosin, one reason for the more efficient inhibition by our protocol is the use of PNB (green) instead of blebbistatin (blue). Another reason may be in the limit of the skinned preparations to totally preserve the OFF-conformation of myosin motors in the absence of Ca^{2+} (49,50).

Supporting Note 3

Modeling the changes of M3 fine structure associated with force step

The axial intensity distribution of the M3 meridional reflection exhibits a fine structure that arises from the X-ray interference between the motor arrays on the two halves of the thick filament and bears information on the

distance between their centers of mass (interference distance, *ID,* Fig. 5A). In the presence of 20 μM PNB, either at rest or during stimulation, the M3 fine structure maintains the characteristic features as in the muscle at rest in physiological solution, being composed of a major peak and two small satellite peaks aside (Fig. S3E and S3H). This feature has been shown to be consistent with the resting conformation of the myosin dimer observed in isolated thick filaments by EM (37, 51).

Following a force step, the overall appearance of M3 fine structure doesn't change (Fig. 4D, left panel), but the relative contributions of the low angle (L_{M3}, Fig. 4M) and high angle (H_{M3}, Fig. 4N) satellite peaks to the M3 vary by detectable amounts, while the spacing of the reflection (S_{M3}) increases (Fig. 4K). These changes occur without changes in the intensity of both the M3 reflection (I_{M3}, Fig. 4L) and the equatorial 1,0 reflection ($I_{1,0}$, Fig. 4E), suggesting that the myosin motors remain in the resting conformation and thus making unlikely that changes in the fine structure of M3 reflection can be attributed, as in previous works (28), to changes in the *ID* due to axial movement of the center of mass of the motors associated with a conformational change that would otherwise change I_{M3}. Here we have tested whether the changes in the M3 fine structure could be explained with the change in the axial periodicity of the myosin motors (responsible for the increase in S_{M3}) being limited to a portion of the array made by the 49 layers of motors (Fig. 1C). Indeed, if the spacing change would occur homogenously throughout the whole thick filament, S_{M3} and *ID* would change by the same relative amount without changes in the M3 fine structure. With the constraint that each myosin dimer remains in the resting conformation, to evaluate its contribution to the M3 each layer of myosin motors can be represented as a point diffractor. The 49 layers are numbered on each half of the thick filament, starting from the one that defines the bare zone (*BZ*, Fig. 5A). The length of *BZ* is taken here as the separation between the centers of mass of the first layer on the two halves of the thick filament.

First, we have considered that, before the force step, in the fiber stimulated in PNB the separation *d* between consecutive layers (Fig. 5A) is constant along each half thick filament and such to give the measured S_{M3}. We have then searched for the *BZ* length that provides the best fit to the observed L_{M3} and H_{M3} by minimizing the squared differences between data and model output (least squares method), while adjusting *d* to fit the observed S_{M3}. With S_{M3} = 14.302 nm, we found *BZ* = 187.9 nm and *d* = 14.299 nm (the slight difference between S_{M3} and *d* is due to the sampling of M3 by the interference function (26)).

The model simulation of the M3 response to the force step predicts that a uniform extension of the whole thick filament, with *BZ* elongating by the same relative amount as the increase in *d* necessary to fit the observed changes of S_{M3}, does not change either L_{M3} or H_{M3} (left and middle panels in Fig. S6K, horizontal dashed lines in Fig. 5B and 5C). This is expected as *ID* (= $BZ + 48 \cdot d$) would also change by the same relative amount as *d* and S_{M3}. The model simulation is repeated first keeping the *BZ* length constant at the value before the step and changing *d* to a different value in the range 14.30-14.45 nm starting from the first layer; then it is repeated keeping also *d* constant at the value before the step in a range between the first and the nth layer (with 1<n<49), and changing it from the nth to the 49th (last) layer. The right panel in Fig. S6K shows the output of the simulation for n = 3. For each value of n the model provides the L_{M3} vs S_{M3} and H_{H3} vs S_{M3} relations (dashed curved lines in Fig. 5B and C respectively for n = 1, 2, 3, 4, 5 and 12). The squared differences between pooled data from the two force steps (0.11 T_0 and 0.22 T_0) and model output have their minimum for n = 3 (L_{M3} vs S_{M3}) and n = 2 (H_{M3} vs S_{M3}), with the overall minimum for n = 2 (Fig. S6L).

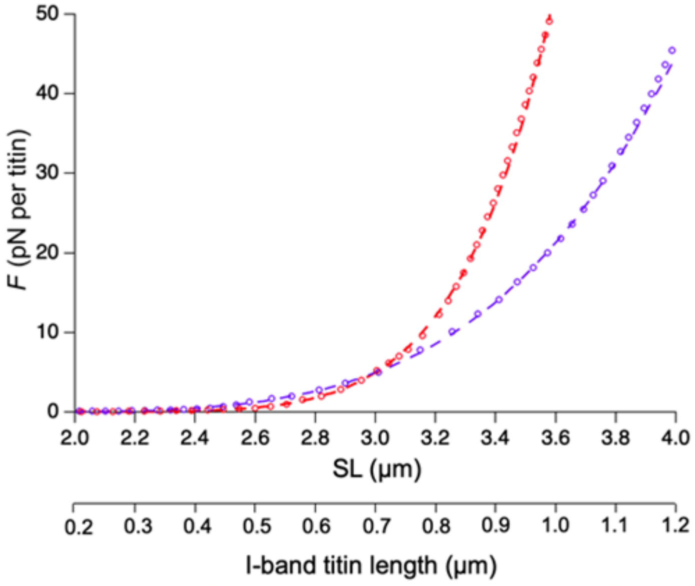

SL (μm)	Spring element	L_p (nm)	I-band length at L_c (nm)	E (pN)
Rat psoas myofibril				
<2.8	tandem-Ig	31 ± 2	660 ± 10	-
>2.8	PEVK	1.31 ± 0.04	1050 ± 80	193 ± 6
Frog muscle fibre				
<2.8	tandem-Ig	42 ± 1	680 ± 30	-
>2.8	PEVK	2.8 ± 0.1	910 ± 40	331± 10

Fig. S1. Passive force-SL relation and its simulation by serially linked tandem Ig and PEVK segments. Dashed lines are the fits to the experimental relations with empirical exponential equations for the frog muscle fiber (red, from Fig. 1B) and rat psoas myofibril (blue, from (21)) with the force expressed in pN per titin molecule. Circles are the result of the simulation obtained assuming the sequential extension of two WLC's, one describing the tandem Ig segment elasticity and the other, integrated by an enthalpic contribution, describing the PEVK segment elasticity. The Table reports the simulation parameters.

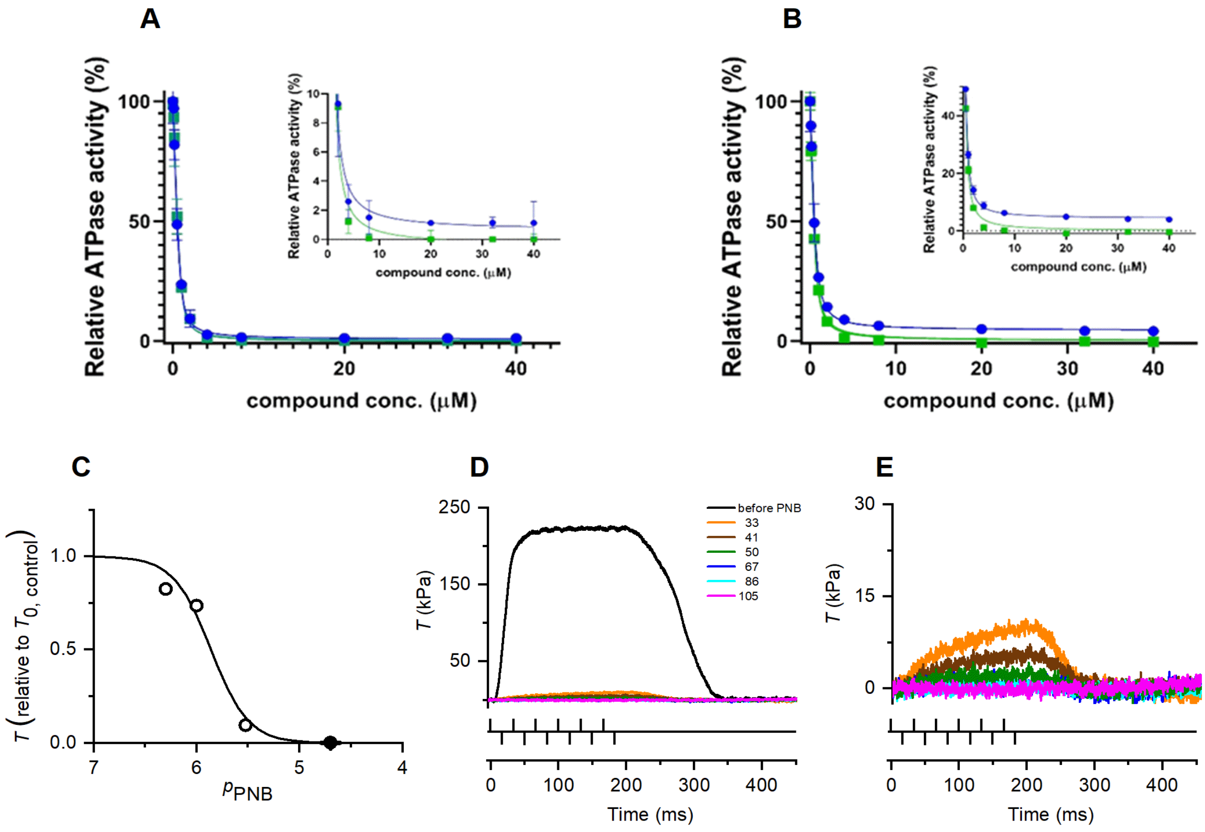

Fig. S2. Stoichiometry and time course of PNB inhibition of frog skeletal muscle myosin. A, B. Titration of the inhibition by PNB (green) and blebbistatin (blue) of the actin-activated ATPase activity of S1 (A) and HMM fragments (B) of myosin extracted from the leg muscles of the frog. Insets are the expanded region of interest of the main figure. IC_{50} values are 0.09 ± 0.03 µM (S1, PNB), 0.10 ± 0.05 µM (S1, blebbistatin), 0.15 ± 0.01 µM (HMM, PNB) and 0.14 ± 0.03 µM (HMM, blebbistatin), maximal ATPase inhibition values are 100 ± 0.5% (S1, PNB), 99.2 ± 0.3% (S1, blebbistatin), 100 ± 0.4% (HMM, PNB) and 95.6 ± 0.9% (HMM, blebbistatin). Data are mean values ± SD from 4 independent measurements for each condition. Temperature 25 °C. C-E. Inhibition of isometric force developed during tetanic stimulation of the frog fiber at 2.15 μm SL and 14 °C. C. Tetanic force developed at the steady state of the effect of PNB at the concentrations indicated in the abscissa (pPNB = -log [PNB]). T is relative to $T_{0,control}$ (the tetanic force developed before PNB perfusion). Open symbols from one fiber of lumbricalis muscle; filled circle mean from all fibers (8 fibers/bundles from lumbricalis and 22 fibers from tibialis anterior). D. Maximum isometric force developed upon tetanic stimulation before (black) and after the start of PNB (20 μM) Ringer perfusion at times (min) identified in the legend by the colors. Single fiber from tibialis anterior. E. Same records as in D following PNB perfusion with the vertical axis expanded 8 times.

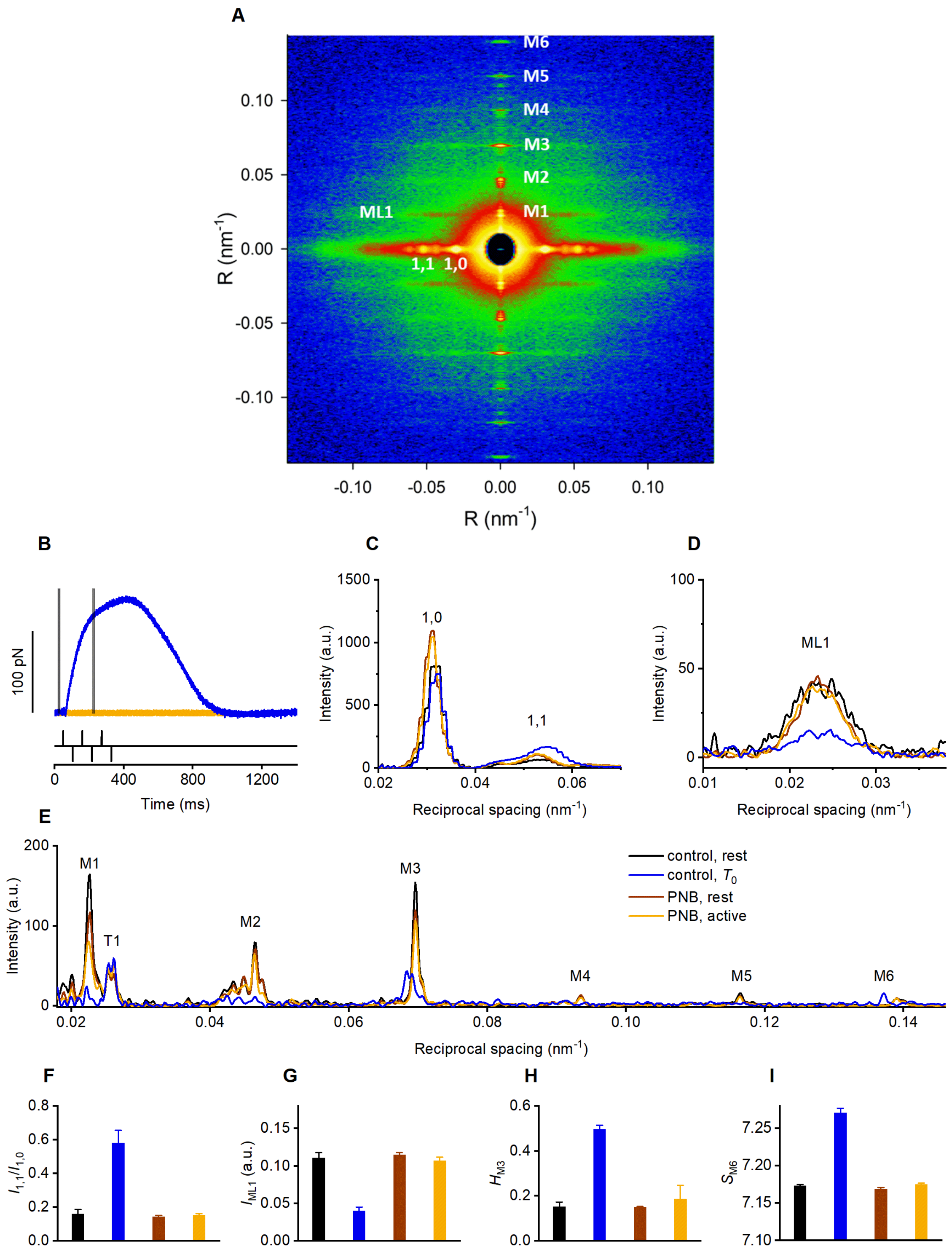

Fig. S3. Structural evidence for 100% inhibition by PNB of myosin filament activation. A. 2D X-ray pattern collected from a single muscle fiber at rest in PNB Ringer with 1.6 m camera length. SL 2.7 µm, temperature 4 °C. Total exposure time 10 ms. The vertical axis is parallel to the fiber axis. B. Force response to tetanic stimulation (lower black trace), either in physiological solution (control, blue) or after the addition of 20 µM PNB (orange). Grey bars: X-ray exposure time windows. The time of active fiber exposure is chosen so as to minimize the effect of the slow creep in force related to the rise of SL inhomogeneity during contractions at SL

above full filament overlap (52). C-E. X-ray diffraction intensity profiles at rest (black, control; brown, PNB) and during tetanic stimulation (blue, control; orange, PNB). Equatorial 1,0 and 1,1 reflections (C), first myosin layer line (ML1, D) and myosin-based meridional reflections, (M1-M6, E). F. Ratio of the intensity of the 1,1 and 1,0 reflections ($I_{1,1}/I_{1,0}$). G. Intensity of ML1 reflection (I_{ML1}). H. Intensity ratio of the high angle peak of the M3 reflection over the total M3 (H_{M3}). I. Spacing of the M6 reflection (S_{M6}). The colors in the F-I histograms identify the same conditions as in C-E. Error bars are SEM. Data in panels F-I are from 8 fibers/bundles, 4 for controls and 4 in PNB; n (rest) = 7 and 11 respectively; n (active) = 8 and 9 respectively.

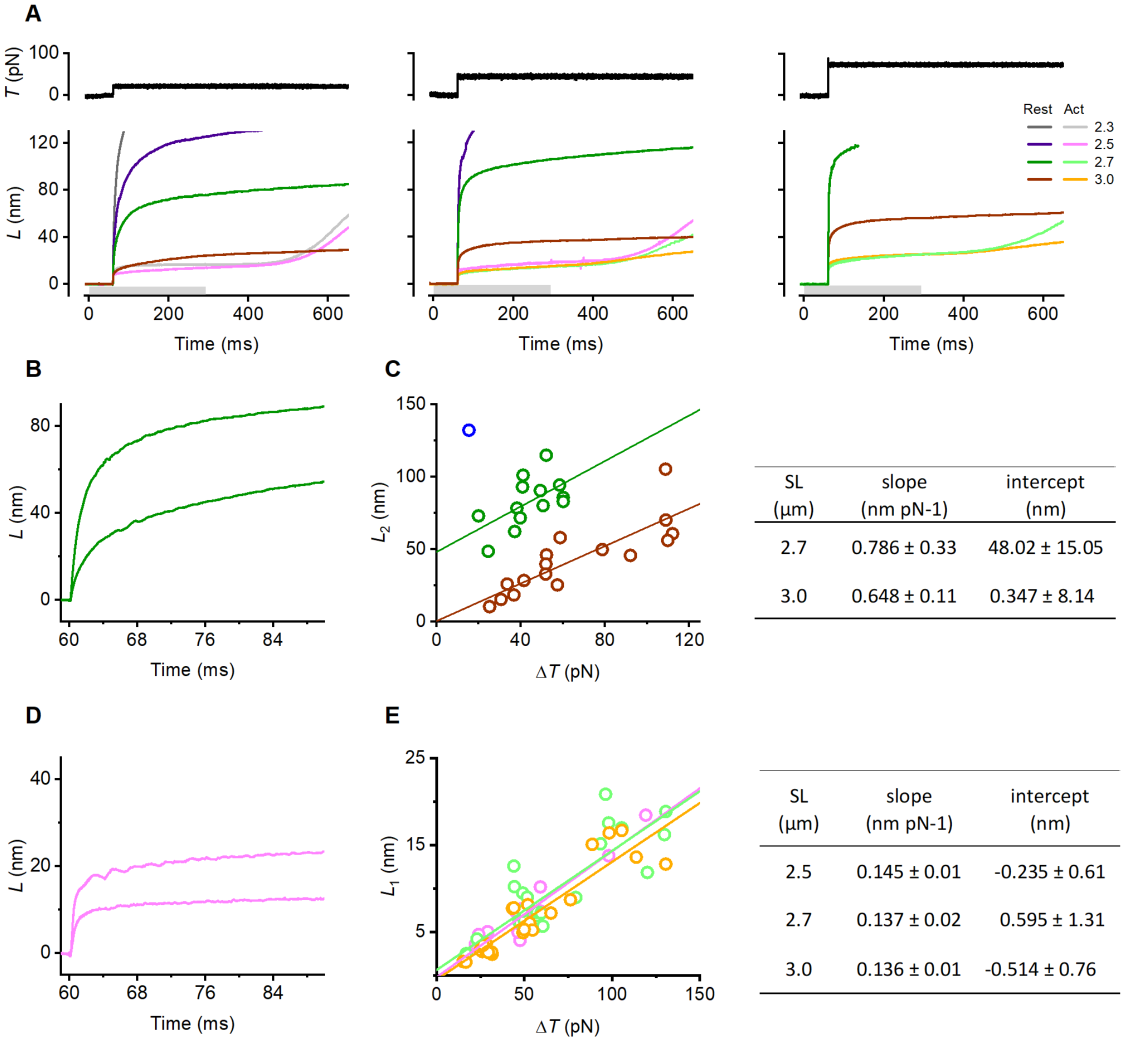

SL (μm)	slope (nm pN-1)	intercept (nm)
2.7	0.786 ± 0.33	48.02 ± 15.05
3.0	0.648 ± 0.11	0.347 ± 8.14

SL (μm)	slope (nm pN-1)	intercept (nm)
2.5	0.145 ± 0.01	-0.235 ± 0.61
2.7	0.137 ± 0.02	0.595 ± 1.31
3.0	0.136 ± 0.01	-0.514 ± 0.76

Fig. S4. Lengthening responses of both resting and stimulated fibers in relation to the SL and the step size. A. Lengthening responses (superimposed traces in each lower panel) to positive force steps (upper traces, black) of 0.13 $T_{0,c}$ (left), 0.20 $T_{0,c}$ (middle) and 0.40 $T_{0,c}$ (right) imposed either at rest (dark colors) or during stimulation (light colors) at different SL: dark and light grey, 2.3 μm; violet and magenta, 2.5 μm; dark and light green, 2.7 μm; brown and orange, 3.0 μm. The same color code, detailed in the inset on the right, where figures indicate the SL (μm), is used throughout panels B-E. Grey horizontal bar: tetanic stimulation. B. Superimposed lengthening responses at 2.7 μm at rest to 0.13 and 0.20 $T_{0,c}$ steps from A (dark green) on a faster time scale. C. Relation between L_2 and ΔT in the resting fiber. Starting SL is 2.5 μm (violet), 2.7 μm (green) and 3.0 μm (brown). At 2.5 μm SL only one response to a small step could be recorded. Lines are the linear fit to data at 2.7 and 3.0 μm SL according to the color code. Data from 6 fibers. The fit parameters are reported in the Table on the right; slopes are not significantly different (P > 0.7). D. Superimposed lengthening responses at 2.5 μm in the active fiber to 0.13 and 0.20 $T_{0,c}$ steps on the same time scale as in B. E. Relation between L_1 and ΔT in the active fiber. Starting SL is 2.5 μm (magenta), 2.7 μm (light green), 3.0 μm (orange).

Lines are the linear fits to data according to the color code. Data from 22 fibers. The fit parameters are reported in the Table on the right; slopes and ordinate intercepts are not significantly different (P always > 0.4).

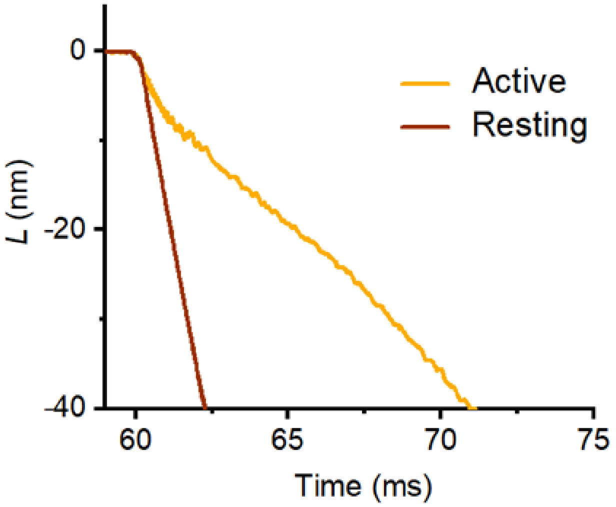

η_{sh} (pN s nm^{-1})	
Rest	Active
$(0.67 \pm 0.21)\cdot 10^{-3}$	$(1.60 \pm 0.35)\cdot 10^{-3}$

Fig. S5 Superimposed shortening responses to a force step of -16 pN imposed at 3 μm SL either at rest (brown) or during stimulation (orange). In the table are reported the viscosity coefficients obtained by linear fit of the $V_{sh} - \Delta T$ data either at rest (5 fibres) or during stimultion (from Table 1) as detailed in Supporting Materials and Methods.

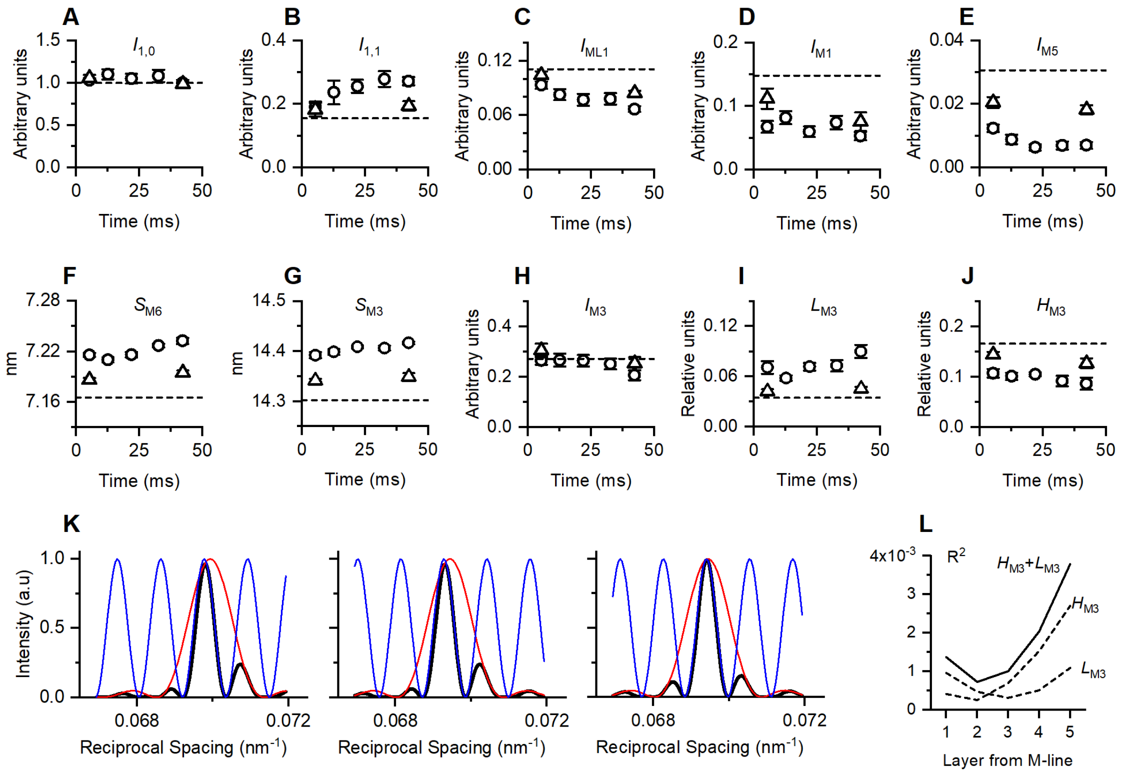

Fig. S6. Dependence of the relevant X-ray signals on the size of the force step and model simulation of the M3 interference fringes. A-E. Intensity of the 1,0 (A), 1,1 (B), ML1 (C), M1 (D) and M5 (E) reflections. F, G. Spacing of the M6 and M3, respectively. H. Intensity of the M3 reflection. I, J. Intensity ratio of the low angle peak (L_{M3}) and of the high angle peak (H_{M3}) respectively over the total M3 intensity. In A-J panels the horizontal dashed line is the value before the step, circles the responses to the 0.22 $T_{0,c}$ step and triangles the responses to the 0.11 $T_{0,c}$ step. Error bars are SEM. Data are from 4 bundles, different number of patterns contributed to different time points as detailed in the legend of Fig. 4. K. Output of the structural model simulation of the effect of a 0.22 $T_{0,c}$ step on the fine structure of the M3 reflection as described in Supporting Note 3. The red profile is the predicted intensity distribution from a single array of motors in the half-thick filament; the blue profile is the intensity distribution from two points separated by a distance equal to *ID*; the black profile is the product of the red and blue profiles and represents the predicted fine structure of the M3 reflection. In the left panel the *BZ* and the motor periodicity *d* are selected for the best fit of the observed intensity profile prior to the force step, with S_{M3}= 14.302 nm. In the central panel S_{M3} has been changed to 14.400 nm with a uniform increase by the same relative amount (14.400/14.302) of both *BZ* and *d*. In the right panel S_{M3} has been changed to 14.400 nm while keeping the same *BZ* and the same *d* up to the 3rd layer of myosin motors as before the force step and changing *d* from the layers 3 to 49. L. Averaged squared residuals (R^2) between the data and the model output as a function of the nth motor layer from which *d* is changed to simulate the data. Dashed lines: R^2 for either L_{M3} vs S_{M3} (Fig. 4M) or H_{M3} vs S_{M3} (Fig. 4N); solid line: R^2 as the sum of the previous two.

SI References

1. A. Magid, D. J. Law, Myofibrils bear most of the resting tension in frog skeletal muscle. *Science* **230**, 1280-1282 (1985).
2. G. Meyer, R. L. Lieber, Muscle fibers bear a larger fraction of passive muscle tension in frogs compared with mice. *J. Exp. Biol.* **221** (2018).
3. A. F. Huxley, V. Lombardi, L. D. Peachey, A system for fast recording of longitudinal displacement of a striated muscle fibre. *J. Physiol. (London)* **317**, 12P-13P (1981).
4. L. Fusi, E. Brunello, M. Reconditi, G. Piazzesi, V. Lombardi, The non-linear elasticity of the muscle sarcomere and the compliance of myosin motors. *J. Physiol. (London)* **592**, 1109-1118 (2014).
5. T. Narayanan *et al.*, A multipurpose instrument for time-resolved ultra-small-angle and coherent X-ray scattering. *J. Appl. Crystallogr.* **51**, 1511-1524 (2018).
6. L. E. Ford, A. F. Huxley, R. M. Simmons, Tension responses to sudden length change in stimulated frog muscle fibres near slack length. *J. Physiol. (London)* **269**, 441-515 (1977).
7. A. F. Huxley, V. Lombardi, A sensitive force transducer with resonant frequency 50 kHz. *J. Physiol. (London)* **305**, 15-16P (1980).
8. V. Lombardi, G. Piazzesi, The contractile response during steady lengthening of stimulated frog muscle fibres. *J. Physiol. (London)* **431**, 141-171 (1990).
9. M. Kepiro *et al.*, para-Nitroblebbistatin, the non-cytotoxic and photostable myosin II inhibitor. *Angew. Chem. Int. Ed. Engl.* **53**, 8211-8215 (2014).
10. M. Gyimesi *et al.*, The mechanism of the reverse recovery step, phosphate release, and actin activation of Dictyostelium myosin II. *J. Biol. Chem.* **283**, 8153-8163 (2008).
11. P. Bianco *et al.* (2014) Fast Force Clamp in Optical Tweezers: A Tool to Study the Kinetics of Molecular Reactions. (Springer Berlin Heidelberg, Berlin, Heidelberg), pp 123-147.
12. J. A. Rivas-Pardo *et al.*, Work Done by Titin Protein Folding Assists Muscle Contraction. *Cell reports* **14**, 1339-1347 (2016).
13. M. Reconditi *et al.*, Sarcomere-length dependence of myosin filament structure in skeletal muscle fibres of the frog. *J. Physiol. (London)* **592**, 1119-1137 (2014).
14. B. A. Mobley, B. R. Eisenberg, Sizes of components in frog skeletal muscle measured by methods of stereology. *J. Gen. Physiol.* **66**, 31-45 (1975).
15. G. Piazzesi, M. Caremani, M. Linari, M. Reconditi, V. Lombardi, Thick Filament Mechano-Sensing in Skeletal and Cardiac Muscles: A Common Mechanism Able to Adapt the Energetic Cost of the Contraction to the Task. *Frontiers in physiology* **9**, 736 (2018).
16. J. C. Haselgrove, X-ray evidence for conformational changes in the myosin filaments of vertebrate striated muscle. *J. Mol. Biol.* **92**, 113-143 (1975).
17. G. Piazzesi *et al.*, Changes in conformation of myosin heads during the development of isometric contraction and rapid shortening in single frog muscle fibres. *J. Physiol. (London)* **514 (Pt 2)**, 305-312 (1999).
18. M.S. Kellermayer, S. B. Smith, H. L. Granzier, C. Bustamante, Folding-unfolding transitions in single titin molecules characterized with laser tweezers. *Science* **276**, 1112-1116 (1997).
19. W. A. Linke, M. R. Stockmeier, M. Ivemeyer, H. Hosser, P. Mundel, Characterizing titin's I-band Ig domain region as an entropic spring. *J. Cell Sci.* **111 (Pt 11)**, 1567-1574 (1998).
20. K. Trombitas *et al.*, Titin extensibility in situ: entropic elasticity of permanently folded and permanently unfolded molecular segments. *J. Cell Biol.* **140**, 853-859 (1998).
21. W. A. Linke, M. Ivemeyer, P. Mundel, M. R. Stockmeier, B. Kolmerer, Nature of PEVK-titin elasticity1in skeletal muscle. *Proc. Natl Acad. Sci. USA* **95**, 8052-8057 (1998).
22. S. Labeit, B. Kolmerer, Titins: giant proteins in charge of muscle ultrastructure and elasticity. *Science* **270**, 293-296 (1995).
23. W. A. Linke *et al.*, Towards a molecular understanding of the elasticity of titin. *J. Mol. Biol.* **261**, 62-71 (1996).
24. J. C. Haselgrove, H. E. Huxley, X-ray evidence for radial cross-bridge movement and for the sliding filament model in actively contracting skeletal muscle. *J. Mol. Biol.* **77**, 549-568 (1973).
25. H. E. Huxley, W. Brown, The low-angle x-ray diagram of vertebrate striated muscle and its behaviour during contraction and rigor. *J. Mol. Biol.* **30**, 383-434 (1967).
26. M. Linari *et al.*, Interference fine structure and sarcomere length dependence of the axial X-ray pattern from active single muscle fibers. *Proc. Natl Acad. Sci. USA* **97**, 7226-7231 (2000).

27. H. E. Huxley, M. Reconditi, A. Stewart, T. Irving, X-ray interference studies of crossbridge action in muscle contraction: evidence from quick releases. *J. Mol. Biol.* **363**, 743-761 (2006).
28. M. Reconditi *et al.*, The myosin motor in muscle generates a smaller and slower working stroke at higher load. *Nature* **428**, 578-581 (2004).
29. P. K. Luther *et al.*, Direct visualization of myosin-binding protein C bridging myosin and actin filaments in intact muscle. *Proc. Natl Acad. Sci. USA* **108**, 11423-11428 (2011).
30. S. B. Malinchik, V. V. Lednev, Interpretation of the X-ray diffraction pattern from relaxed skeletal muscle and modelling of the thick filament structure. *J. Muscle Res. Cell Motil.* **13**, 406-419 (1992).
31. K. Oshima *et al.*, Axial dispositions and conformations of myosin crossbridges along thick filaments in relaxed and contracting states of vertebrate striated muscles by X-ray fiber diffraction. *J. Mol. Biol.* **367**, 275-301 (2007).
32. E. Rome, G. Offer, F. A. Pepe, X-ray diffraction of muscle labelled with antibody to C-protein. *Nat New Biol* **244**, 152-154 (1973).
33. J. M. Squire, "X-ray diffraction methods in muscle research" in The Structural Basis of Muscular Contraction. (Plenum Press, New York, 1981), pp. 39.
34. C. Moos, C. M. Mason, J. M. Besterman, I. N. Feng, J. H. Dubin, The binding of skeletal muscle C-protein to F-actin, and its relation to the interaction of actin with myosin subfragment-1. *J. Mol. Biol.* **124**, 571-586 (1978).
35. J. M. Squire, P. K. Luther, C. Knupp, Structural evidence for the interaction of C-protein (MyBP-C) with actin and sequence identification of a possible actin-binding domain. *J. Mol. Biol.* **331**, 713-724 (2003).
36. M. Reconditi *et al.*, Motion of myosin head domains during activation and force development in skeletal muscle. *Proc. Natl Acad. Sci. USA* **108**, 7236-7240 (2011).
37. J. L. Woodhead *et al.*, Atomic model of a myosin filament in the relaxed state. *Nature* **436**, 1195-1199 (2005).
38. I. Dobbie *et al.*, Elastic bending and active tilting of myosin heads during muscle contraction. *Nature* **396**, 383-387 (1998).
39. G. Piazzesi *et al.*, Skeletal muscle performance determined by modulation of number of myosin motors rather than motor force or stroke size. *Cell* **131**, 784-795 (2007).
40. G. Piazzesi *et al.*, Mechanism of force generation by myosin heads in skeletal muscle. *Nature* **415**, 659-662 (2002).
41. E. Brunello *et al.*, The contributions of filaments and cross-bridges to sarcomere compliance in skeletal muscle. *J. Physiol. (London)* **592**, 3881-3899 (2014).
42. M. Linari *et al.*, Force generation by skeletal muscle is controlled by mechanosensing in myosin filaments. *Nature* **528**, 276-279 (2015).
43. W. Ma, H. Gong, T. Irving, Myosin Head Configurations in Resting and Contracting Murine Skeletal Muscle. *Int J Mol Sci* **19** (2018).
44. M. Reconditi *et al.*, Thick Filament Length Changes in Muscle Have Both Elastic and Structural Components. *Biophys. J.* **116**, 983-984 (2019).
45. N. Yagi, An x-ray diffraction study on early structural changes in skeletal muscle contraction. *Biophys. J.* **84**, 1093-1102 (2003).
46. T. Matsuo, N. Yagi, Structural changes in the muscle thin filament during contractions caused by single and double electrical pulses. *J. Mol. Biol.* **383**, 1019-1036 (2008).
47. L. Fusi, E. Brunello, Z. Yan, M. Irving, Thick filament mechano-sensing is a calcium-independent regulatory mechanism in skeletal muscle. *Nature communications* **7**, 13281 (2016).
48. H. Iwamoto, Effects of myosin inhibitors on the X-ray diffraction patterns of relaxed and calcium-activated rabbit skeletal muscle fibers. *Biophys Physicobiol* **15**, 111-120 (2018).
49. M. Caremani *et al.*, Inotropic interventions do not change the resting state of myosin motors during cardiac diastole. *J. Gen. Physiol.* **151**, 53-65 (2019).
50. M. Caremani *et al.*, Dependence of thick filament structure in relaxed mammalian skeletal muscle on temperature and interfilament spacing. *J. Gen. Physiol.* **153** (2021).
51. M. E. Zoghbi, J. L. Woodhead, R. L. Moss, R. Craig, Three-dimensional structure of vertebrate cardiac muscle myosin filaments. *Proc. Natl Acad. Sci. USA* **105**, 2386-2390 (2008).
52. A. M. Gordon, A. F. Huxley, F. J. Julian, The variation in isometric tension with sarcomere length in vertebrate muscle fibres. *J. Physiol. (London)* **184**, 170-192 (1966).
